# Analyzing chaos and superposition of lump waves with other waves in the time-fractional coupled nonlinear schördinger equation

**DOI:** 10.1371/journal.pone.0304334

**Published:** 2024-08-28

**Authors:** Sheikh Zain Majid, Muhammad Imran Asjad, Muhammad Bilal Riaz, Taseer Muhammad

**Affiliations:** 1 Department of Mathematics, University of Management and Technology, Lahore, Pakistan; 2 Department of Computer Science and Mathematics, Lebanese American University, Byblos, Lebanon; 3 It4innovations, VSB-Technical University of Ostrava, Ostrava, Czech Republic; 4 Department of Mathematics, College of Science, King Khalid University, Abha, Saudi Arabia; Institute of Space Technology, PAKISTAN

## Abstract

This article aims to study the time fractional coupled nonlinear Schrödinger equation, which explains the interaction between modes in nonlinear optics and Bose-Einstein condensation. The proposed generalized projective Riccati equation method and modified auxiliary equation method extract a more efficient and broad range of soliton solutions. These include novel solutions like a combined dark-lump wave soliton, multiple dark-lump wave soliton, two dark-kink solitons, flat kink-lump wave, multiple U-shaped with lump wave, combined bright-dark with high amplitude lump wave, bright-dark with lump wave and kink dark-periodic solitons are derived. The travelling wave patterns of the model are graphically presented with suitable parameters in 3D, density, contour and 2D surfaces, enhancing understanding of parameter impact. The proposed model’s dynamics were observed and presented as quasi-periodic chaotic, periodic systems and quasi-periodic. This analysis confirms the effectiveness and reliability of the method employed, demonstrating its applicability in discovering travelling wave solitons for a wide range of nonlinear evolution equations.

## 1 Introduction

Nonlinear partial differential equations (PDEs) are necessary to study nonlinear physical settings. The Schrödinger-type governing equation is one of these significant equations and a special tool for understanding intricate nonlinear structures. This equation has important applications in many scientific and engineering areas, such as mathematical physics, optics, fibre optics, plasma physics, and communication engineering [1]. Finding accurate and dependable soliton solutions for the nonlinear Schrödinger equation, which describes the system’s mechanical features, is one of the most important in practical mathematics. Nonlinear PDEs have gained increasing importance and popularity in mathematical theory and various applications over the past few decades [2]. Schrödinger-type equations have practical applications and are fundamental in many domains, including mathematical physics, plasma research, and telecommunications engineering [3]. The range of scientific applications available to mathematicians has grown with the rapid development of technology and computer programs. It is impossible to exaggerate the importance of PDEs since they provide a practical way to simulate various engineering and natural phenomena. In applied sciences and mathematics, precise solutions to these equations are essential to understanding the physical characteristics of nonlinear systems [4–8].

This study is dedicated to the examination of a time-fractional coupled nonlinear Schrödinger equation (TFCNSE) in (1 + 1)-dimensions, incorporating conformable fractional derivatives [9–11].

$$\begin{array}{c} \begin{array}{c} \iota \;D_{t}^{\ell }\psi_{1}+D_{t}^{2\beta }\psi_{1}+ℵ\left({\left|\psi_{1}\right|}^{2}+\Omega {\left|\psi_{2}\right|}^{2}\right)\psi_{1}=0,\\ \iota \;D_{t}^{\ell }\psi_{2}+D_{t}^{2\beta }\psi_{1}+ℵ\left({\left|\psi_{1}\right|}^{2}+\Omega {\left|\psi_{2}\right|}^{2}\right)\psi_{2}=0. \end{array} \end{array}$$ (1)

The given conditions and functions are part of a mathematical model describing circulatory-polarised waves’ behaviour in nonlinear optical fibres. The parameters 0 < *ℓ*, *β* ≤ 1, have specific constraints, and the functions *ψ*_1_(*x*, *t*) and *ψ*_2_(*x*, *t*) characterize the amplitude of these waves at different spatial and temporal components. The variables *x* and *t* denote spatial and temporal coordinates, respectively. Additionally, Ω and ℵ are real numbers that play a role in this description. $\iota \;D_{t}^{\ell }\psi_{1},\;D_{t}^{2\beta }\psi_{1}$ represent the conformable fractional derivative operators with respect to *t* and *x* replacing $\iota \;D_{t}^{\ell }\psi_{1}$ and $D_{t}^{2\beta }\psi_{1}$ with ∂_*t*_ and ∂_*x*_, respectively, and letting *ℓ* = 1, the TFCNSE Eq (1) is the integer-order equation [12]. The nonlinear model allows for analysing nonlinear effects in optical fibres under the given conditions and functions.

The discovery of solitary wave propagation by John Scott Russell in the 1870s gives rise to the concept of solitons. Before Russell’s discovery, distinguished scientists and philosophers knew the possible consequences of solitons. Boussinesq and Rayleigh’s contributions emphasised the significance of nonlinearity and dispersion in comprehending solitons. The problems raised by Airy and Stokes regarding modelling travelling wave phenomena in important domains such as elastic media and fibre optics using soliton solutions are currently under investigation. [13–16] discusses two well-known solutions: the Boussinesq equation and the Korteweg-de Vries travelling wave solutions. The research aims to develop better wave propagation forecasts in these domains by developing solutions that more closely reflect the real world.

The analysis and derivation of solutions to numerous nonlinear equations have become fundamentally dependent on travelling solitary waves. Recently, scientists and experts have stressed the importance of finding exact standalone solutions. These kinds of solutions are essential to comprehending the behaviour and stability of physical systems. In addition, some solitary solutions, such as soliton, quasi-periodic, periodic, rational, and coupon solutions, have been found for nonlinear evolution equations (NLEEs). Nonlinear PDEs find extensive applications in soliton wave theory, including mass and hydrodynamics, plasma physics, heat transport, and chemical engineering [17, 18]. Moreover, nonlinear ordinary differential equation systems play a crucial role in describing complex biological processes [19], population ecology [20], electromagnetic wave-plasma interactions [21], different phenomena in quantum mechanics that have been studied in [22, 23].

Numerous methodologies are employed to approach soliton solutions, ranging from traditional techniques to modern computational methods, including the combined elliptic Jacobian functions [24], the sine-Gordon expansion technique [25], the bilinear neural network method [26], new extended direct algebraic method [27], neural network Levenberg-Marquardt [28], modified Sardar sub-equation method [29] and numerous others [30–36].

Numerous researchers have focused on studying the complex nonlinear integrable wave Eq (1). Ahmed et al. [37] used the modified version of the extended tanh-expansion scheme and discovered some general soliton solutions. In [38], Ali et al. used the F-modified expansion and unified techniques and obtained a U-shaped soliton. The q-homotopy analysis transform method is applied to obtain the analytical solutions [39], semi-inverse variational principle method and trial equation method [40]. The methods proposed in this study are more efficient and reliable in constructing travelling wave solutions than current analytical methods. This implies that the discussed techniques provide advantages over existing methods in accuracy, ease of use, and potential computational efficiency. These details could include comparative analyses, numerical experiments, or specific examples that showcase how the proposed technique outperforms the alternatives. This would provide evidence to substantiate the claims about its advantages and effectiveness.

The TFCNSE equation model, which is a coupled nonlinear Schrödinger equation with time-fractional derivatives, has not been extensively analyzed using the Generalized projective Riccati equation (GPRE) and Modified auxiliary equation (MAE) methods. Additionally, the aspects of chaos theory analysis have been neglected. This study uses GPRE and MAE methods to discover more stable and generalized soliton solutions. Our current methodologies offer several advantages over previously used methods, providing a more generalized and efficient soliton solution. These solutions encompass various types of novel soliton solutions like combined dark-lump wave soliton, Multiple dark-lump wave soliton, two dark-kink soliton, flat kink-lump wave, multiple U-shaped with lump wave, combined bright-dark with high amplitude lump wave, bright-dark with lump wave and kink dark-periodic solitons are derived. The solutions of the TFCNLSE are expressed in both trigonometric and hyperbolic functions to represent their mathematical nature. We use the Maple software to provide graphical representations of the solutions for visual comparison. We depict the solutions diagrammatically by varying the parameter values, providing a comprehensive visualization of their behaviour and characteristics. By implementing the GPRE and MAE methods and the graphical analysis of the solutions, this study contributes to understanding soliton dynamics in the TFCNSE. The presented methodology offers a more generalized and effective approach for obtaining soliton solutions in this context. Obtained solutions can be essential in understanding the behaviour of waves in nonlinear media, utilised in fibre optics and the manipulation of laser beams, and contribute to our understanding of complex systems in physics. Finally, the considered equation is studied dynamically using chaos theory and bifurcation analysis on the developed new dynamical system. We expect our innovative discoveries to significantly contribute to the communication industry and ocean engineering, offering valuable insights to engineers and physicists that can generate new ideas.

Section 2 focused on developing precise solutions using the GPRE and MAE methods. Section 3 then demonstrated how this method can be effectively employed. Additionally, we explored the influence of wave velocity on the visual representation of soliton solutions in various graphical formats, including 3D plots, density plots, contour plots, and 2D pictorial representations. These findings are further explained in Section 4, which includes a comparative study and discussion. Section 5 delved into the chaotic behaviour exhibited by the considered model, presenting our observations through graphical analysis. Finally, Section 6 encompasses the original study conclusion.

## 2 Description of the proposed techniques

Various methodologies can be applied to derive the soliton solution for Eq (1). Let’s start by considering a general nonlinear PDE expressed in the following form:

$$\begin{array}{c} Y(\Xi ,\;\Xi_{t},\;\Xi_{x},\;\Xi_{xt},\;\Xi_{xx},\cdots )=0, \end{array}$$ (2)

In the provided Eq (2), the term *Ξ*(*x*, *t*) denotes a polynomial containing the highest order derivative and nonlinear term. Using a transformation methodology makes it feasible to convert Eq (2) into an ODE.

$$\begin{array}{c} \Xi (x,t)=\Pi (\eta ),\;\eta =x\;-c\;t, \end{array}$$ (3)

$$\begin{array}{c} Q(\Pi ,\Pi^{\prime },\Pi^{\prime \prime },\ldots )=0, \end{array}$$ (4)

where *c*, *Q* and $\Pi^{\prime }=\frac{d\;\Pi }{d\;\eta }$ are velocity can be represented as a polynomial concerning Π(*η*), respectively.

### 2.1 Generalized projective Riccati equation method

**Step 1**. Assuming the solution of Eq (4) in accordance with the discussion in [41],

$$\begin{array}{c} \Pi \left(\eta \right)=A_{0}+\sum_{i=1}^{W}Z^{i-1}\left(\eta \right)[A_{i}Z\left(\eta \right)+C_{i}M\left(\eta \right)], \end{array}$$ (5)

arbitrary constants in Eq (5) denote as *A*_0_, *A*_*i*_, and *C*_*i*_ (where *i* = 1, …, *W*). The functions *Z*(*η*) and *M*(*η*) satisfy ODEs.

$$\begin{array}{c} \begin{array}{cc} & M^{\prime }\left(\eta \right)=\delta +\epsilon \;M^{2}\left(\eta \right)-\vartheta \;M(\eta ),\;\epsilon =\pm 1,\\ & Z^{\prime }\left(\eta \right)=\epsilon \;M\left(\eta \right)\;Z\left(\eta \right), \end{array} \end{array}$$ (6)

such that,

$$\begin{array}{c} M^{2}\left(\eta \right)=-\epsilon [\delta -2\;\epsilon^{2}\vartheta \;Z(\eta )+\frac{\vartheta^{2}\epsilon^{2}{\left(Z(\eta )\right)}^{2}}{\delta }-\frac{\epsilon^{2}{\left(Z(\eta )\right)}^{2}}{\delta }], \end{array}$$ (7)

where *ϑ* and *δ* are non zero constants.

If *ϑ* = *δ* = 0 in Eq (6),

$$\begin{array}{c} \Pi \left(\eta \right)=\sum_{i=1}^{W}A_{i}M^{i}\left(\eta \right), \end{array}$$ (8)

where *M*^*i*^(*η*) satisfies the nonlinear ODE,

$$\begin{array}{c} M^{\prime }\left(\eta \right)=M^{2}\left(\eta \right). \end{array}$$ (9)

**Step 2**. The determination of the positive integer *W* in Eq (8) involves calculating it through a homogeneous balance principle of the highest nonlinear term and the terms with the highest-order derivatives, as expressed in Eq (4).

**Step 3**. By inserting Eq (8) along with Eqs (6) and (7) into Eq (4), we can obtain a system of algebraic equations. Then, we can organize the terms of *Z*^*j*^(*η*) and *M*^*i*^(*η*) based on their respective orders (where *j* = 0, 1, 2, … and *i* = 0, 1). By setting all coefficients of these equations, we can get the desired values of parameters.

**Step 4**. The solutions for Eq (6) can be found in [42]. They are provided below.

(Set 1) if *ϵ* = −1, *δ* ≠ 0,

$$\begin{array}{c} \begin{array}{cc} & Z_{1}\left(x,t\right)=\frac{\delta \;\text{sech}(\sqrt{\delta }\;\eta )}{\vartheta \;\text{sech}(\sqrt{\delta }\;\eta )+1},\;M_{1}\left(x,t\right)=\frac{\sqrt{\delta }\;\text{tanh}\left(\sqrt{\delta }\;\eta \right)}{\vartheta \;\text{sech}(\sqrt{\delta }\;\eta )+1}, \end{array} \end{array}$$ (10)

$$\begin{array}{c} \begin{array}{cc} & Z_{2}\left(x,t\right)=\frac{\delta \;\text{csch}(\sqrt{\delta }\;\eta )}{\vartheta \;\text{csch}(\sqrt{\delta }\;\eta )+1},\;M_{2}\left(x,t\right)=\frac{\sqrt{\delta }\;\text{coth}\left(\sqrt{\delta }\;\eta \right)}{\vartheta \;\text{csch}(\sqrt{\delta }\;\eta )+1}, \end{array} \end{array}$$ (11)

(Set 2) if *ϵ* = 1, *δ* ≠ 0,

$$\begin{array}{c} \begin{array}{cc} & Z_{3}\left(x,t\right)=\frac{\delta \;\text{sec}(\sqrt{\delta }\;\eta )}{\vartheta \;\text{sec}(\sqrt{\delta }\;\eta )+1},\;M_{3}\left(x,t\right)=\frac{\sqrt{\delta }\;\text{tan}\left(\sqrt{\delta }\;\eta \right)}{\vartheta \;\text{sec}(\sqrt{\delta }\;\eta )+1}, \end{array} \end{array}$$ (12)

$$\begin{array}{c} \begin{array}{cc} & Z_{4}\left(x,t\right)=\frac{\delta \;\text{csc}(\sqrt{\delta }\;\eta )}{\vartheta \;\text{csc}(\sqrt{\delta }\;\eta )+1},\;M_{4}\left(x,t\right)=\frac{\sqrt{\delta }\;\text{cot}\left(\sqrt{\delta }\;\eta \right)}{\vartheta \;\text{csc}(\sqrt{\delta }\;\eta )+1}, \end{array} \end{array}$$ (13)

(Set 3) if *ϑ* = *δ* = 0,

$$\begin{array}{c} Z_{5}\left(x,t\right)=\frac{c_{1}}{\eta },\;M_{5}\left(x,t\right)=\frac{1}{\epsilon \;\eta }, \end{array}$$ (14)

where *c*_1_ denotes the constant parameter.

**Step 5**. By substituting solutions obtained in Eqs (10)–(14) and the parameter values into Eq (5), we can obtain the soliton solutions for Eq (2).

### 2.2 Modified auxiliary equation method

**Step 1**. To clarify, we can assume the solution of Eq (4) based on the discussion presented in [43].

$$\begin{array}{c} \Pi \left(\eta \right)=A_{0}+\sum_{i=1}^{W}[A_{i}{\left(z^{h(\eta )}\right)}^{i}+C_{i}{\left(z^{-h(\eta )}\right)}^{i}], \end{array}$$ (15)

arbitrary constants in this equation are denoted as *A*_0_, *A*_*i*_, and *C*_*i*_ (where *i* = 1, …, *W*), while the function *h*(*η*) follows the auxiliary equation.

$$\begin{array}{c} h^{\prime }\left(\eta \right)=\frac{\nu +\kappa \;z^{-h(\eta )}+\zeta \;z^{h(\eta )}}{\text{ln}(z)}, \end{array}$$ (16)

where *κ*, *ν* and *ζ* are non zero constants with *z* > 0, *z* ≠ 1, further *A*_0_, *B*_*i*_ and *C*_*i*_, are cannot be zero simultaneously.

**Step 2**. The positive integer *W* in Eq (15) is determined by a homogeneous balance of the highest nonlinear term and the highest-order derivative terms in Eq (4).

**Step 3**. By substituting the equation represented as Eq (15), along with Eq (16), into Eq (4), we can obtain a system of the algebraic equations by gathering terms of different powers of the *z*^*h*(*η*)^. We can solve the system of equations by setting all coefficients to zero and determining the desired values of the parameters.

**Step 4**. The proposed solutions for the Eq (16) are provided as follows [44]:

(Set 1) if *ν*^2^−4*κζ* < 0 and *ζ* ≠ 0,

$$\begin{array}{c} \begin{array}{ccc} & z^{h(\eta )}=\frac{-\nu +\sqrt{4\kappa \;\zeta -\nu^{2}}\;\text{tan}\left(\frac{\sqrt{4\kappa \;\zeta -\nu^{2}}\;\eta }{2}\right)}{2\;\zeta }\;\;\;or\; & z^{h(\eta )}=\frac{-\nu +\sqrt{4\kappa \;\zeta -\nu^{2}}\;\text{cot}\left(\frac{\sqrt{4\kappa \;\zeta -\nu^{2}}\;\eta }{2}\right)}{2\;\zeta } \end{array} \end{array}$$ (17)

(Set 2) if *ν*^2^ − 4*κζ* > 0 and *ζ* ≠ 0,

$$\begin{array}{c} \begin{array}{ccc} & z^{h(\eta )}=-\frac{\nu +\sqrt{\nu^{2}-4\kappa \;\zeta }\;\text{tan}\left(\frac{\sqrt{\nu^{2}-4\kappa \;\zeta }\;\eta }{2}\right)}{2\;\zeta }\;\;\;or\; & z^{h(\eta )}=-\frac{\nu +\sqrt{\nu^{2}-4\kappa \;\zeta }\;\text{cot}\left(\frac{\sqrt{\nu^{2}-4\kappa \;\zeta }\;\eta }{2}\right)}{2\;\zeta } \end{array} \end{array}$$ (18)

(Set 3) if *ν*^2^ − 4*κζ* = 0 and *ζ* ≠ 0,

$$\begin{array}{c} \begin{array}{c} z^{h(\eta )}=-\frac{2+\nu \;\eta }{2\;\zeta \;\eta } \end{array} \end{array}$$ (19)

Step 5. By substituting solutions obtained in Eqs (17)–(19) and the parameter values into Eq (15), we can obtain the soliton solutions for Eq (2).

## 3 Application of purposed methods

To find the exact soliton solutions of the time-fractional coupled nonlinear Schrödinger equation equation, the wave transformation applied to the system (1),

$$\begin{array}{c} \phi_{1}\left(x,t\right)=\psi_{1}\left(\eta \right)\;e^{\iota \theta } \end{array}$$ (20)

$$\begin{array}{c} \phi_{2}\left(x,t\right)=\psi_{2}\left(\eta \right)\;e^{\iota \theta } \end{array}$$ (21)

and

$$\begin{array}{c} \eta =r(\frac{x^{\beta }}{\beta }-v\frac{t^{\ell }}{\ell }),\;\;\theta =-\sigma \frac{x^{\beta }}{\beta }+\omega \frac{t^{\ell }}{\ell }+\Gamma , \end{array}$$ (22)

where *r*, *σ* and *ω* are the real constants and Γ represents the arbitrary constant where *β* and *ℓ* are the fractional operators at the conditions 0 < *ℓ*, *β* ≤ 1. By substituting the complex wave transformation (20) and (21) into (1) we get,

$$\begin{array}{c} \left\{ \begin{array}{c} r^{2}\psi_{1}^{\prime \prime }+ℵ\psi_{1}^{3}+ℵ\Omega \psi_{2}^{2}\psi_{1}-\left(\sigma^{2}+\omega \right)\psi_{1}=0,\\ r^{2}\psi_{2}^{\prime \prime }+ℵ\psi_{2}^{3}+ℵ\Omega \psi_{1}^{2}\psi_{2}-\left(\sigma^{2}+\omega \right)\psi_{2}=0. \end{array}\right. \end{array}$$ (23)

By decomposing the real and imaginary parts we get *c* = −2*σ*.

By assuming,

$$\begin{array}{c} \psi_{2}=f\psi_{1}. \end{array}$$ (24)

Then Eq (23) becomes,

$$\begin{array}{c} r^{2}\psi_{1}^{\prime \prime }+\left(ℵ+ℵ\;\Omega \;f^{2}\right)\psi_{1}^{3}-\left(\sigma^{2}+\omega \right)\psi_{1}=0 \end{array}$$ (25)

### 3.1 Exact solutions for the GPRE method with graphical representation

It has been determined by using the rule of homogeneous balancing between the terms $\psi_{1}^{\prime \prime }$ and $\psi_{1}^{3}$ of Eq (25), implies, *W* + 2 = 3*W*, we get *W* = 1. The GPRE method can be used to find the general soliton solutions of the model.

$$\begin{array}{c} \psi \left(\eta \right)=A_{0}+A_{1}\;Z(\eta )+C_{1}\;M(\eta ), \end{array}$$ (26)

Substituting the system of Eq (26) along with Eqs (6) and (7) into Eq (25) and comparing the coefficients of the different powers of *Z*(*η*) and *M*(*η*), we get an algebraic system.

$$\begin{array}{c} \begin{array}{cc} Z{\left(\eta \right)}^{0}M{\left(\eta \right)}^{0}= & \;3\;\delta \Omega \;ℵ\;f^{2}\epsilon \;A_{0}{C_{1}}^{2}+\Omega \;ℵ\;f^{2}{A_{0}}^{3}-3\;\delta ℵ\;\epsilon \;A_{0}{C_{1}}^{2}+ℵ\;{A_{0}}^{3}-\sigma^{2}A_{0}-\omega \;A_{0}=0,\\ Z{\left(\eta \right)}^{0}M{\left(\eta \right)}^{1}= & -\delta \Omega \;ℵ\;f^{2}\epsilon \;{C_{1}}^{3}+3\;\Omega \;ℵ\;f^{2}{A_{0}}^{2}C_{1}-\delta ℵ\;\epsilon \;{C_{1}}^{3}+3\;ℵ\;{b_{0}}^{2}C_{1}-\sigma^{2}C_{1}-\omega \;C_{1}=0,\\ Z{\left(\eta \right)}^{1}M{\left(\eta \right)}^{0}= & -3\;\delta \Omega \;ℵ\;f^{2}\epsilon \;A_{1}{C_{1}}^{2}+6\;\Omega \;ℵ\;f^{2}\vartheta \;\epsilon \;A_{0}{C_{1}}^{2}-2\;\delta r^{2}\epsilon^{3}A_{1}+3\;\Omega \;ℵ\;f^{2}{A_{0}}^{2}A_{1}\\ & -3\;\delta ℵ\;\epsilon \;A_{1}{C_{1}}^{2}+6\;ℵ\;\vartheta \;\epsilon \;b_{0}{C_{1}}^{2}+\delta r^{2}\epsilon \;A_{1}+3\;ℵ\;{A_{0}}^{2}b_{1}-\sigma^{2}A_{1}-\omega \;A_{1}=0,\\ Z{\left(\eta \right)}^{1}M{\left(\eta \right)}^{1}= & \;2\;\Omega \;ℵ\;f^{2}\vartheta \;\epsilon \;{C_{1}}^{3}+6\;\Omega \;ℵ\;f^{2}A_{0}A_{1}C_{1}+2\;\vartheta \;r^{2}\epsilon^{3}b_{2}+2\;ℵ\;\vartheta \;\epsilon \;{C_{1}}^{3}-\vartheta \;r^{2}\epsilon \;b_{2}\\ & +6\;ℵ\;A_{0}A_{1}C_{1}=0,\\ Z{\left(\eta \right)}^{2}M{\left(\eta \right)}^{0}= & -\;\frac{3\;A_{0}{C_{1}}^{2}ℵ\;\Omega \;f^{2}\vartheta^{2}\epsilon }{\delta }-r^{2}A_{1}\epsilon \;\vartheta +4\;r^{2}A_{1}\epsilon^{3}\vartheta +3\;A_{0}{A_{1}}^{2}ℵ+3\;A_{0}{A_{1}}^{2}ℵ\;\Omega \;f^{2}\\ & +\frac{3\;A_{0}{C_{1}}^{2}ℵ\;\epsilon }{\delta }+6\;A_{1}{C_{1}}^{2}ℵ\;\vartheta \;\epsilon +\frac{3\;A_{0}{C_{1}}^{2}ℵ\;\Omega \;f^{2}\epsilon }{\delta }+6\;A_{1}{C_{1}}^{2}ℵ\;\Omega \;f^{2}\vartheta \;\epsilon \\ & -\frac{3\;A_{0}{C_{1}}^{2}ℵ\;\vartheta^{2}\epsilon }{\delta }=0,\\ Z{\left(\eta \right)}^{2}M{\left(\eta \right)}^{1}= & -\frac{3\;A_{0}{C_{1}}^{2}ℵ\;\Omega \;f^{2}\vartheta^{2}\epsilon }{\delta }-r^{2}A_{1}\epsilon \;\vartheta +4\;r^{2}A_{1}\epsilon^{3}\vartheta +3\;A_{0}{A_{1}}^{2}ℵ+3\;A_{0}{A_{1}}^{2}ℵ\;\Omega \;f^{2}\\ & +\frac{3\;A_{0}{C_{1}}^{2}ℵ\;\epsilon }{\delta }+6\;A_{1}{C_{1}}^{2}ℵ\;\vartheta \;\epsilon +\frac{3\;A_{0}{C_{1}}^{2}ℵ\;\Omega \;f^{2}\epsilon }{\delta }+6\;A_{1}{C_{1}}^{2}ℵ\;\Omega \;f^{2}\vartheta \;\epsilon \\ & -\frac{3\;A_{0}{C_{1}}^{2}ℵ\;\vartheta^{2}\epsilon }{\delta }=0,\\ Z{\left(\eta \right)}^{3}= & -\frac{3\;A_{1}{C_{1}}^{2}ℵ\;\Omega \;f^{2}\vartheta^{2}\epsilon }{\delta }+2\;\frac{r^{2}A_{1}\epsilon^{3}}{\delta }+{A_{1}}^{3}ℵ+{A_{1}}^{3}ℵ\;\Omega \;f^{2}+\frac{3\;A_{1}{b_{2}}^{2}ℵ\;\epsilon }{\delta }-2\;\frac{r^{2}A_{1}\epsilon^{3}\vartheta^{2}}{\delta }\\ & +\frac{3\;A_{1}{C_{1}}^{2}ℵ\;\Omega \;f^{2}\epsilon }{\delta }-\frac{3\;A_{1}{C_{1}}^{2}ℵ\;\vartheta^{2}\epsilon }{\delta }. \end{array} \end{array}$$ (27)

the modern software called Mathematica. The results of the solution are now available.

**Case-1**:

$$\begin{array}{c} \begin{array}{cc} & \omega =-\frac{\iota }{2}\delta r^{2}\sqrt{2}-\sigma^{2},\;\epsilon =\pm \sqrt{\frac{-1}{2}},\;A_{0}=A_{0},\;A_{1}=0,\;f=f,\\ & C_{1}=\sqrt{(\Omega \;ℵ\;f^{2}+ℵ{)}^{-1}}r. \end{array} \end{array}$$ (28)

**Case-2**:

$$\begin{array}{c} \begin{array}{cc} & \omega =\frac{1}{2}\;\delta r^{2}-\sigma^{2},\;\epsilon =1,\;A_{0}=0,\;A_{1}=\pm \sqrt{-\frac{-\vartheta^{2}+1}{2\;\delta \Omega \;ℵ\;f^{2}+2\;\delta ℵ}},\;f=f,\\ & C_{1}=\pm \sqrt{-(2\;\Omega \;ℵ\;f^{2}+2\;ℵ{)}^{-1}}r. \end{array} \end{array}$$ (29)

**Case-3**:

$$\begin{array}{c} \begin{array}{cc} & \omega =-\frac{1}{2}\;\delta r^{2}-\sigma^{2},\;\epsilon =-1,\;A_{0}=0,\;A_{1}=\pm \sqrt{-\frac{\vartheta^{2}-1}{2\;\delta \Omega \;ℵ\;f^{2}+2\;\delta ℵ}}r,\;f=f,\\ & C_{1}=\sqrt{-(2\;\Omega \;ℵ\;f^{2}+2\;ℵ{)}^{-1}}r. \end{array} \end{array}$$ (30)

**Case-4**:

$$\begin{array}{c} \begin{array}{cc} & f=\pm \sqrt{-\Omega^{-1}},\;\omega =-\sigma^{2},\;\epsilon =0,\;A_{0}=A_{0},\;A_{1}=A_{1},\;C_{1}=C_{1}. \end{array} \end{array}$$ (31)

To obtain solutions for Case 1, we can substitute Eq (28) to into Eq (26) and then we have,

(Set 3) if *ϑ* = *δ* = 0,

$$\begin{array}{c} \psi_{1,1}\left(x,t\right)=(A_{0}+\sqrt{(\Omega \;ℵ\;f^{2}+ℵ{)}^{-1}}r\times \frac{\sqrt{2}}{\sqrt{-1}\;\eta })\times \;e^{\iota (\sigma \frac{x^{\beta }}{\beta }+\left(-\frac{\iota }{2}\delta r^{2}\sqrt{2}-\sigma^{2}\right)\frac{t^{\ell }}{\ell }+\Gamma )}. \end{array}$$ (32)

put this value in (24), we get,

$$\begin{array}{c} \psi_{1,2}\left(x,t\right)=f\;(A_{0}+\sqrt{(\Omega \;ℵ\;f^{2}+ℵ{)}^{-1}}r\times \frac{\sqrt{2}}{\sqrt{-1}\;\eta })\times \;e^{\iota (\sigma \frac{x^{\beta }}{\beta }+\left(-\frac{\iota }{2}\delta r^{2}\sqrt{2}-\sigma^{2}\right)\frac{t^{\ell }}{\ell }+\Gamma )}. \end{array}$$ (33)

To obtain solutions for **Case 2**, we can follow the same procedure: substitute Eq (29) into Eq (26), we get,

(Set 2): if *ϵ* = 1, *δ* ≠ 0,

$$\begin{array}{c} \begin{array}{cc} \psi_{2,1}\left(x,t\right)= & (\pm \sqrt{-\frac{-\vartheta^{2}+1}{2\;\delta \Omega \;ℵ\;f^{2}+2\;\delta ℵ}}\times \frac{\delta \;\text{sec}(\sqrt{\delta }\;\eta )}{\vartheta \;\text{sec}(\sqrt{\delta }\;\eta )+1}\\ & \pm \sqrt{-(2\;\Omega \;ℵ\;f^{2}+2\;ℵ{)}^{-1}}r\times \frac{\sqrt{\delta }\;\text{tan}\left(\sqrt{\delta }\;\eta \right)}{\vartheta \;\text{sec}(\sqrt{\delta }\;\eta )+1})\times \;e^{\iota (\sigma \frac{x^{\beta }}{\beta }+\left(\frac{1}{2}\;\delta r^{2}-\sigma^{2}\right)\frac{t^{\ell }}{\ell }+\Gamma )}. \end{array} \end{array}$$ (34)

$$\begin{array}{c} \begin{array}{cc} \psi_{2,2}\left(x,t\right)= & f(\pm \sqrt{-\frac{-\vartheta^{2}+1}{2\;\delta \Omega \;ℵ\;f^{2}+2\;\delta ℵ}}\times \frac{\delta \;\text{sec}(\sqrt{\delta }\;\eta )}{\vartheta \;\text{sec}(\sqrt{\delta }\;\eta )+1}\\ & \pm \sqrt{-(2\;\Omega \;ℵ\;f^{2}+2\;ℵ{)}^{-1}}r\times \frac{\sqrt{\delta }\;\text{tan}\left(\sqrt{\delta }\;\eta \right)}{\vartheta \;\text{sec}(\sqrt{\delta }\;\eta )+1})\times \;e^{\iota (\sigma \frac{x^{\beta }}{\beta }+\left(\frac{1}{2}\;\delta r^{2}-\sigma^{2}\right)\frac{t^{\ell }}{\ell }+\Gamma )}. \end{array} \end{array}$$ (35)

$$\begin{array}{c} \begin{array}{cc} \psi_{3,1}\left(x,t\right)= & (\pm \sqrt{-\frac{-\vartheta^{2}+1}{2\;\delta \Omega \;ℵ\;f^{2}+2\;\delta ℵ}}\times \frac{\delta \;\text{csc}(\sqrt{\delta }\;\eta )}{\vartheta \;\text{csc}(\sqrt{\delta }\;\eta )+1}\\ & \pm \sqrt{-(2\;\Omega \;ℵ\;f^{2}+2\;ℵ{)}^{-1}}r\times \frac{\sqrt{\delta }\;\text{cot}\left(\sqrt{\delta }\;\eta \right)}{\vartheta \;\text{csc}(\sqrt{\delta }\;\eta )+1})\times \;e^{\iota (\sigma \frac{x^{\beta }}{\beta }+\left(\frac{1}{2}\;\delta r^{2}-\sigma^{2}\right)\frac{t^{\ell }}{\ell }+\Gamma )}. \end{array} \end{array}$$ (36)

$$\begin{array}{c} \begin{array}{cc} \psi_{3,2}\left(x,t\right)= & f\;(\pm \sqrt{-\frac{-\vartheta^{2}+1}{2\;\delta \Omega \;ℵ\;f^{2}+2\;\delta ℵ}}\times \frac{\delta \;\text{csc}(\sqrt{\delta }\;\eta )}{\vartheta \;\text{csc}(\sqrt{\delta }\;\eta )+1}\\ & \pm \sqrt{-(2\;\Omega \;ℵ\;f^{2}+2\;ℵ{)}^{-1}}r\times \frac{\sqrt{\delta }\;\text{cot}\left(\sqrt{\delta }\;\eta \right)}{\vartheta \;\text{csc}(\sqrt{\delta }\;\eta )+1})\times \;e^{\iota (\sigma \frac{x^{\beta }}{\beta }+\left(\frac{1}{2}\;\delta r^{2}-\sigma^{2}\right)\frac{t^{\ell }}{\ell }+\Gamma )}. \end{array} \end{array}$$ (37)

(Set 3): if *ϑ* = *δ* = 0,

$$\begin{array}{c} \begin{array}{cc} \psi_{4,1}\left(x,t\right)= & (\pm \sqrt{-\frac{-\vartheta^{2}+1}{2\;\delta \Omega \;ℵ\;f^{2}+2\;\delta ℵ}}\times \frac{c_{1}}{\eta }\pm \sqrt{-(2\;\Omega \;ℵ\;f^{2}+2\;ℵ{)}^{-1}}r\times \frac{1}{\eta })\times \\ & e^{\iota (\sigma \frac{x^{\beta }}{\beta }+\left(\frac{1}{2}\;\delta r^{2}-\sigma^{2}\right)\frac{t^{\ell }}{\ell }+\Gamma )}. \end{array} \end{array}$$ (38)

$$\begin{array}{c} \begin{array}{cc} \psi_{4,2}\left(x,t\right)= & f\;(\pm \sqrt{-\frac{-\vartheta^{2}+1}{2\;\delta \Omega \;ℵ\;f^{2}+2\;\delta ℵ}}\times \frac{c_{1}}{\eta }\pm \sqrt{-(2\;\Omega \;ℵ\;f^{2}+2\;ℵ{)}^{-1}}r\times \frac{1}{\eta })\times \\ & e^{\iota (\sigma \frac{x^{\beta }}{\beta }+\left(\frac{1}{2}\;\delta r^{2}-\sigma^{2}\right)\frac{t^{\ell }}{\ell }+\Gamma )}. \end{array} \end{array}$$ (39)

To derive solutions for Case 3, we proceed by substituting Eq (30) into Eq (26).

(Set 1): if *ϵ* = −1, *δ* ≠ 0,

$$\begin{array}{c} \begin{array}{cc} \psi_{5,1}\left(x,t\right)= & (\pm \sqrt{-\frac{\vartheta^{2}-1}{2\;\delta \Omega \;ℵ\;f^{2}+2\;\delta ℵ}}r\times \frac{\delta \;\text{sech}(\sqrt{\delta }\;\eta )}{\vartheta \;\text{sech}(\sqrt{\delta }\;\eta )+1}\\ & \pm \sqrt{-(2\;\Omega \;ℵ\;f^{2}+2\;ℵ{)}^{-1}}r\times \frac{\sqrt{\delta }\;\text{tanh}\left(\sqrt{\delta }\;\eta \right)}{\vartheta \;\text{sech}(\sqrt{\delta }\;\eta )+1})\times \;e^{\iota (\sigma \frac{x^{\beta }}{\beta }+\left(\frac{1}{2}\;\delta r^{2}-\sigma^{2}\right)\frac{t^{\ell }}{\ell }+\Gamma )}. \end{array} \end{array}$$ (40)

$$\begin{array}{c} \begin{array}{cc} \psi_{5,2}\left(x,t\right)= & f\;(\pm \sqrt{-\frac{\vartheta^{2}-1}{2\;\delta \Omega \;ℵ\;f^{2}+2\;\delta ℵ}}r\times \frac{\delta \;\text{sech}(\sqrt{\delta }\;\eta )}{\vartheta \;\text{sech}(\sqrt{\delta }\;\eta )+1}\\ & \pm \sqrt{-(2\;\Omega \;ℵ\;f^{2}+2\;ℵ{)}^{-1}}r\times \frac{\sqrt{\delta }\;\text{tanh}\left(\sqrt{\delta }\;\eta \right)}{\vartheta \;\text{sech}(\sqrt{\delta }\;\eta )+1})\times \;e^{\iota (\sigma \frac{x^{\beta }}{\beta }+\left(\frac{1}{2}\;\delta r^{2}-\sigma^{2}\right)\frac{t^{\ell }}{\ell }+\Gamma )}. \end{array} \end{array}$$ (41)

$$\begin{array}{c} \begin{array}{cc} \psi_{6,1}\left(x,t\right)= & (\pm \sqrt{-\frac{\vartheta^{2}-1}{2\;\delta \Omega \;ℵ\;f^{2}+2\;\delta ℵ}}r\times \frac{\delta \;\text{csch}(\sqrt{\delta }\;\eta )}{\vartheta \;\text{csch}(\sqrt{\delta }\;\eta )+1}\\ & \pm \sqrt{-(2\;\Omega \;ℵ\;f^{2}+2\;ℵ{)}^{-1}}r\times \frac{\sqrt{\delta }\;\text{coth}\left(\sqrt{\delta }\;\eta \right)}{\vartheta \;\text{csch}(\sqrt{\delta }\;\eta )+1})\times \;e^{\iota (\sigma \frac{x^{\beta }}{\beta }+\left(\frac{1}{2}\;\delta r^{2}-\sigma^{2}\right)\frac{t^{\ell }}{\ell }+\Gamma )}. \end{array} \end{array}$$ (42)

$$\begin{array}{c} \begin{array}{cc} \psi_{6,2}\left(x,t\right)= & f\;(\pm \sqrt{-\frac{\vartheta^{2}-1}{2\;\delta \Omega \;ℵ\;f^{2}+2\;\delta ℵ}}r\times \frac{\delta \;\text{csch}(\sqrt{\delta }\;\eta )}{\vartheta \;\text{csch}(\sqrt{\delta }\;\eta )+1}\\ & \pm \sqrt{-(2\;\Omega \;ℵ\;f^{2}+2\;ℵ{)}^{-1}}r\times \frac{\sqrt{\delta }\;\text{coth}\left(\sqrt{\delta }\;\eta \right)}{\vartheta \;\text{csch}(\sqrt{\delta }\;\eta )+1})\times \;e^{\iota (\sigma \frac{x^{\beta }}{\beta }+\left(\frac{1}{2}\;\delta r^{2}-\sigma^{2}\right)\frac{t^{\ell }}{\ell }+\Gamma )}. \end{array} \end{array}$$ (43)

(Set 3): if *ϑ* = *δ* = 0,

$$\begin{array}{c} \begin{array}{cc} \psi_{7,1}\left(x,t\right)= & (\pm \sqrt{-\frac{\vartheta^{2}-1}{2\;\delta \Omega \;ℵ\;f^{2}+2\;\delta ℵ}}r\times \frac{c_{1}}{\eta }\pm \sqrt{-(2\;\Omega \;ℵ\;f^{2}+2\;ℵ{)}^{-1}}r\times \frac{1}{\eta })\times \\ & e^{\iota (\sigma \frac{x^{\beta }}{\beta }+\left(\frac{1}{2}\;\delta r^{2}-\sigma^{2}\right)\frac{t^{\ell }}{\ell }+\Gamma )}. \end{array} \end{array}$$ (44)

$$\begin{array}{c} \begin{array}{cc} \psi_{7,2}\left(x,t\right)= & f\;(\pm \sqrt{-\frac{\vartheta^{2}-1}{2\;\delta \Omega \;ℵ\;f^{2}+2\;\delta ℵ}}r\times \frac{c_{1}}{\eta }\pm \sqrt{-(2\;\Omega \;ℵ\;f^{2}+2\;ℵ{)}^{-1}}r\times \frac{1}{\eta })\times \\ & e^{\iota (\sigma \frac{x^{\beta }}{\beta }+\left(\frac{1}{2}\;\delta r^{2}-\sigma^{2}\right)\frac{t^{\ell }}{\ell }+\Gamma )}. \end{array} \end{array}$$ (45)

In **Case 4**, when the value of *ϵ* = 0, it leads to division by zero in the equation, which renders the solution undefined.

### 3.2 Exact solutions for the MAE with method graphical representation

It has been determined by using the rule of homogeneous balancing between the terms $\psi_{1}^{\prime \prime }$ and $\psi_{1}^{3}$ of Eq (25), we get, *W* = 1. The MAE method can be used to find the general soliton solutions of the model.

$$\begin{array}{c} \psi \left(\eta \right)=A_{0}+A_{1}z^{h(\eta )}+C_{1}z^{-h(x)}, \end{array}$$ (46)

Substituting the system of Eq (46) into Eq (25) and comparing the coefficients of the different powers of *z*^*h*(*η*)^, we get an algebraic system.

$$\begin{array}{c} \begin{array}{cc} z^{0h(\eta )}= & \Omega \;ℵ\;f^{2}{A_{0}}^{3}+6\;\Omega \;ℵ\;f^{2}A_{0}A_{1}C_{1}+\kappa \;\nu \;r^{2}A_{1}+\nu \;\zeta \;r^{2}C_{1}+ℵ\;{A_{0}}^{3}+6\;ℵ\;A_{0}A_{1}C_{1}\\ & -\sigma^{2}A_{0}-\omega \;A_{0},\\ z^{h(\eta )}= & 3\;\Omega \;ℵ\;f^{2}{A_{0}}^{2}A_{1}+3\;\Omega \;ℵ\;f^{2}{A_{1}}^{2}C_{1}+2\;\kappa \;\zeta \;r^{2}A_{1}+\nu^{2}r^{2}A_{1}+3\;ℵ\;{A_{0}}^{2}A_{1}+3\;ℵ\;{A_{1}}^{2}C_{1}\\ & -\sigma^{2}A_{1}-\omega \;A_{1},\\ z^{2h(\eta )}= & 3\;\Omega \;ℵ\;f^{2}A_{0}{A_{1}}^{2}+3\;\nu \;\zeta \;r^{2}A_{1}+3\;ℵ\;A_{0}{A_{1}}^{2},\\ z^{3h(\eta )}= & \Omega \;ℵ\;f^{2}{A_{1}}^{3}+2\;\zeta^{2}r^{2}A_{1}+ℵ\;{A_{1}}^{3},\\ z^{-h(\eta )}= & 3\;\Omega \;ℵ\;f^{2}{A_{0}}^{2}C_{1}+3\;\Omega \;ℵ\;f^{2}A_{1}{C_{1}}^{2}+2\;\kappa \;\zeta \;r^{2}C_{1}+\nu^{2}r^{2}C_{1}+3\;ℵ\;{A_{0}}^{2}C_{1}+3\;ℵ\;A_{1}{C_{1}}^{2}\\ & -\sigma^{2}C_{1}-\omega \;C_{1},\\ z^{-2h(\eta )}= & 3\;\Omega \;ℵ\;f^{2}A_{0}{C_{1}}^{2}+3\;\kappa \;\nu \;r^{2}C_{1}+3\;ℵ\;A_{0}{C_{1}}^{2},\\ z^{-3h(\eta )}= & \Omega \;ℵ\;f^{2}{C_{1}}^{3}+2\;\kappa^{2}r^{2}C_{1}+ℵ\;{C_{1}}^{3}. \end{array} \end{array}$$ (47)

The system mentioned in Eq (47) was solved using the modern software called Mathematica. The results of the solution are now available.

**Case-1**:

$$\begin{array}{c} \begin{array}{cc} & \sigma =\sigma ,\;\omega =2\;\kappa \;\zeta \;r^{2}-\frac{1}{2}\;\nu^{2}r^{2}-\sigma^{2},\;A_{0}=-\frac{\nu \;r}{ℵ\;(\Omega \;f^{2}+1)}\frac{1}{\sqrt{-2\;(\Omega \;ℵ\;f^{2}+ℵ{)}^{-1}}}\\ & A_{1}=\zeta \;\sqrt{-2\;(\Omega \;ℵ\;f^{2}+ℵ{)}^{-1}}r,\;C_{1}=0,\;r=r. \end{array} \end{array}$$ (48)

**Case-2**:

$$\begin{array}{c} \begin{array}{cc} & \sigma =\sigma ,\;\omega =2\;\kappa \;\zeta \;r^{2}-\frac{1}{2}\;\nu^{2}r^{2}-\sigma^{2},\;A_{0}=-\frac{\nu \;r}{ℵ\;(\Omega \;f^{2}+1)}\frac{1}{\sqrt{-2\;(\Omega \;ℵ\;f^{2}+ℵ{)}^{-1}}}\\ & A_{1}=0,\;C_{1}=\sqrt{-2\;(\Omega \;ℵ\;f^{2}+ℵ{)}^{-1}}r\kappa ,\;r=r. \end{array} \end{array}$$ (49)

To obtain solutions for **Case 1**, where we introduce Eq (48) into Eq (46), proceed as follows:

(Set 1) if *ν*^2^ − 4*κζ* < 0 and *ζ* ≠ 0,

$$\begin{array}{c} \begin{array}{cc} \psi_{8,1}\left(x,t\right)= & (-\frac{\nu \;r}{ℵ\;(\Omega \;f^{2}+1)}\frac{1}{\sqrt{-2\;(\Omega \;ℵ\;f^{2}+ℵ{)}^{-1}}}+\zeta \;\sqrt{-2\;(\Omega \;ℵ\;f^{2}+ℵ{)}^{-1}}r\\ & \times (\frac{-\nu +\sqrt{4\kappa \;\zeta -\nu^{2}}\;\text{tan}\left(\frac{\sqrt{4\kappa \;\zeta -\nu^{2}}\;\eta }{2}\right)}{2\;\zeta }))\times \;e^{\iota (\sigma \frac{x^{\beta }}{\beta }+\left(2\;\kappa \;\zeta \;r^{2}-\frac{1}{2}\;\nu^{2}r^{2}-\sigma^{2}\right)\frac{t^{\ell }}{\ell }+\Gamma )}. \end{array} \end{array}$$ (50)

put this value in Eq (24), we get,

$$\begin{array}{c} \begin{array}{cc} \psi_{8,2}\left(x,t\right)= & f(-\frac{\nu \;r}{ℵ\;(\Omega \;f^{2}+1)}\frac{1}{\sqrt{-2\;(\Omega \;ℵ\;f^{2}+ℵ{)}^{-1}}}+\zeta \;\sqrt{-2\;(\Omega \;ℵ\;f^{2}+ℵ{)}^{-1}}r\\ & \times (\frac{-\nu +\sqrt{4\kappa \;\zeta -\nu^{2}}\;\text{tan}\left(\frac{\sqrt{4\kappa \;\zeta -\nu^{2}}\;\eta }{2}\right)}{2\;\zeta }))\times \;e^{\iota (\sigma \frac{x^{\beta }}{\beta }+\left(2\;\kappa \;\zeta \;r^{2}-\frac{1}{2}\;\nu^{2}r^{2}-\sigma^{2}\right)\frac{t^{\ell }}{\ell }+\Gamma )}. \end{array} \end{array}$$ (51)

**Or**

$$\begin{array}{c} \begin{array}{cc} \psi_{9,1}\left(x,t\right)= & (-\frac{\nu \;r}{ℵ\;(\Omega \;f^{2}+1)}\frac{1}{\sqrt{-2\;(\Omega \;ℵ\;f^{2}+ℵ{)}^{-1}}}+\zeta \;\sqrt{-2\;(\Omega \;ℵ\;f^{2}+ℵ{)}^{-1}}r\\ & \times (\frac{-\nu +\sqrt{4\kappa \;\zeta -\nu^{2}}\text{cot}\left(\frac{\sqrt{4\kappa \;\zeta -\nu^{2}}\;\eta }{2}\right)}{2\;\zeta }))\times \;e^{\iota (\sigma \frac{x^{\beta }}{\beta }+\left(2\;\kappa \;\zeta \;r^{2}-\frac{1}{2}\;\nu^{2}r^{2}-\sigma^{2}\right)\frac{t^{\ell }}{\ell }+\Gamma )}. \end{array} \end{array}$$ (52)

$$\begin{array}{c} \begin{array}{cc} \psi_{9,2}\left(x,t\right)= & f(-\frac{\nu \;r}{ℵ\;(\Omega \;f^{2}+1)}\frac{1}{\sqrt{-2\;(\Omega \;ℵ\;f^{2}+ℵ{)}^{-1}}}+\zeta \;\sqrt{-2\;(\Omega \;ℵ\;f^{2}+ℵ{)}^{-1}}r\\ & \times (\frac{-\nu +\sqrt{4\kappa \;\zeta -\nu^{2}}\text{cot}\left(\frac{\sqrt{4\kappa \;\zeta -\nu^{2}}\;\eta }{2}\right)}{2\;\zeta }))\times \;e^{\iota (\sigma \frac{x^{\beta }}{\beta }+\left(2\;\kappa \;\zeta \;r^{2}-\frac{1}{2}\;\nu^{2}r^{2}-\sigma^{2}\right)\frac{t^{\ell }}{\ell }+\Gamma )}. \end{array} \end{array}$$ (53)

(Set 2) if *ν*^2^ − 4*κζ* > 0 and *ζ* ≠ 0,

$$\begin{array}{c} \begin{array}{cc} \psi_{10,1}\left(x,t\right)= & (-\frac{\nu \;r}{ℵ\;(\Omega \;f^{2}+1)}\frac{1}{\sqrt{-2\;(\Omega \;ℵ\;f^{2}+ℵ{)}^{-1}}}+\zeta \;\sqrt{-2\;(\Omega \;ℵ\;f^{2}+ℵ{)}^{-1}}r\\ & \times (-\frac{\nu +\sqrt{\nu^{2}-4\kappa \;\zeta }\;\text{tan}\left(\frac{\sqrt{\nu^{2}-4\kappa \;\zeta }\;\eta }{2}\right)}{2\;\zeta }))\times \;e^{\iota (\sigma \frac{x^{\beta }}{\beta }+\left(2\;\kappa \;\zeta \;r^{2}-\frac{1}{2}\;\nu^{2}r^{2}-\sigma^{2}\right)\frac{t^{\ell }}{\ell }+\Gamma )}. \end{array} \end{array}$$ (54)

$$\begin{array}{c} \begin{array}{cc} \psi_{10,2}\left(x,t\right)= & f(-\frac{\nu \;r}{ℵ\;(\Omega \;f^{2}+1)}\frac{1}{\sqrt{-2\;(\Omega \;ℵ\;f^{2}+ℵ{)}^{-1}}}+\zeta \;\sqrt{-2\;(\Omega \;ℵ\;f^{2}+ℵ{)}^{-1}}r\\ & \times (-\frac{\nu +\sqrt{\nu^{2}-4\kappa \;\zeta }\;\text{tan}\left(\frac{\sqrt{\nu^{2}-4\kappa \;\zeta }\;\eta }{2}\right)}{2\;\zeta }))\times \;e^{\iota (\sigma \frac{x^{\beta }}{\beta }+\left(2\;\kappa \;\zeta \;r^{2}-\frac{1}{2}\;\nu^{2}r^{2}-\sigma^{2}\right)\frac{t^{\ell }}{\ell }+\Gamma )}. \end{array} \end{array}$$ (55)

**Or**

$$\begin{array}{c} \begin{array}{cc} \psi_{11,1}\left(x,t\right)= & (-\frac{\nu \;r}{ℵ\;(\Omega \;f^{2}+1)}\frac{1}{\sqrt{-2\;(\Omega \;ℵ\;f^{2}+ℵ{)}^{-1}}}+\zeta \;\sqrt{-2\;(\Omega \;ℵ\;f^{2}+ℵ{)}^{-1}}r\\ & \times (-\frac{\nu +\sqrt{\nu^{2}-4\kappa \;\zeta }\text{cot}\left(\frac{\sqrt{\nu^{2}-4\kappa \;\zeta }\;\eta }{2}\right)}{2\;\zeta }))\times \;e^{\iota (\sigma \frac{x^{\beta }}{\beta }+\left(2\;\kappa \;\zeta \;r^{2}-\frac{1}{2}\;\nu^{2}r^{2}-\sigma^{2}\right)\frac{t^{\ell }}{\ell }+\Gamma )}. \end{array} \end{array}$$ (56)

$$\begin{array}{c} \begin{array}{cc} \psi_{11,2}\left(x,t\right)= & f(-\frac{\nu \;r}{ℵ\;(\Omega \;f^{2}+1)}\frac{1}{\sqrt{-2\;(\Omega \;ℵ\;f^{2}+ℵ{)}^{-1}}}+\zeta \;\sqrt{-2\;(\Omega \;ℵ\;f^{2}+ℵ{)}^{-1}}r\\ & \times (-\frac{\nu +\sqrt{\nu^{2}-4\kappa \;\zeta }\text{cot}\left(\frac{\sqrt{\nu^{2}-4\kappa \;\zeta }\;\eta }{2}\right)}{2\;\zeta }))\times \;e^{\iota (\sigma \frac{x^{\beta }}{\beta }+\left(2\;\kappa \;\zeta \;r^{2}-\frac{1}{2}\;\nu^{2}r^{2}-\sigma^{2}\right)\frac{t^{\ell }}{\ell }+\Gamma )}. \end{array} \end{array}$$ (57)

(Set 3) if *ν*^2^ − 4*κζ* = 0 and *ζ* ≠ 0,

$$\begin{array}{c} \begin{array}{cc} \psi_{12,1}\left(x,t\right)= & (-\frac{\nu \;r}{ℵ\;(\Omega \;f^{2}+1)}\frac{1}{\sqrt{-2\;(\Omega \;ℵ\;f^{2}+ℵ{)}^{-1}}}+\zeta \;\sqrt{-2\;(\Omega \;ℵ\;f^{2}+ℵ{)}^{-1}}r\\ & \times (-\frac{2+\nu \;\eta }{2\;\zeta \;\eta }))\times \;e^{\iota (\sigma \frac{x^{\beta }}{\beta }+\left(2\;\kappa \;\zeta \;r^{2}-\frac{1}{2}\;\nu^{2}r^{2}-\sigma^{2}\right)\frac{t^{\ell }}{\ell }+\Gamma )}. \end{array} \end{array}$$ (58)

$$\begin{array}{c} \begin{array}{cc} \psi_{12,2}\left(x,t\right)= & f(-\frac{\nu \;r}{ℵ\;(\Omega \;f^{2}+1)}\frac{1}{\sqrt{-2\;(\Omega \;ℵ\;f^{2}+ℵ{)}^{-1}}}+\zeta \;\sqrt{-2\;(\Omega \;ℵ\;f^{2}+ℵ{)}^{-1}}r\\ & \times (-\frac{2+\nu \;\eta }{2\;\zeta \;\eta }))\times \;e^{\iota (\sigma \frac{x^{\beta }}{\beta }+\left(2\;\kappa \;\zeta \;r^{2}-\frac{1}{2}\;\nu^{2}r^{2}-\sigma^{2}\right)\frac{t^{\ell }}{\ell }+\Gamma )}. \end{array} \end{array}$$ (59)

To obtain solutions for Case 2, where we introduce Eq (49) into Eq (46), we proceed as follows:

(Set 1) if *ν*^2^ − 4*κζ* < 0 and *ζ* ≠ 0,

$$\begin{array}{c} \begin{array}{cc} \psi_{13,1}\left(x,t\right)= & (-\frac{\nu \;r}{ℵ\;(\Omega \;f^{2}+1)}\frac{1}{\sqrt{-2\;(\Omega \;ℵ\;f^{2}+ℵ{)}^{-1}}}+\;\sqrt{-2\;(\Omega \;ℵ\;f^{2}+ℵ{)}^{-1}}r\;\kappa \\ & \times (\frac{-\nu +\sqrt{4\kappa \;\zeta -\nu^{2}}\;\text{tan}\left(\frac{\sqrt{4\kappa \;\zeta -\nu^{2}}\;\eta }{2}\right)}{2\;\zeta }{)}^{-1})\times \;e^{\iota (\sigma \frac{x^{\beta }}{\beta }+\left(2\;\kappa \;\zeta \;r^{2}-\frac{1}{2}\;\nu^{2}r^{2}-\sigma^{2}\right)\frac{t^{\ell }}{\ell }+\Gamma )}. \end{array} \end{array}$$ (60)

$$\begin{array}{c} \begin{array}{cc} \psi_{13,2}\left(x,t\right)= & f(-\frac{\nu \;r}{ℵ\;(\Omega \;f^{2}+1)}\frac{1}{\sqrt{-2\;(\Omega \;ℵ\;f^{2}+ℵ{)}^{-1}}}+\;\sqrt{-2\;(\Omega \;ℵ\;f^{2}+ℵ{)}^{-1}}r\;\kappa \\ & \times (\frac{-\nu +\sqrt{4\kappa \;\zeta -\nu^{2}}\;\text{tan}\left(\frac{\sqrt{4\kappa \;\zeta -\nu^{2}}\;\eta }{2}\right)}{2\;\zeta }{)}^{-1})\times \;e^{\iota (\sigma \frac{x^{\beta }}{\beta }+\left(2\;\kappa \;\zeta \;r^{2}-\frac{1}{2}\;\nu^{2}r^{2}-\sigma^{2}\right)\frac{t^{\ell }}{\ell }+\Gamma )}. \end{array} \end{array}$$ (61)

**Or**

$$\begin{array}{c} \begin{array}{cc} \psi_{14,1}\left(x,t\right)= & (-\frac{\nu \;r}{ℵ\;(\Omega \;f^{2}+1)}\frac{1}{\sqrt{-2\;(\Omega \;ℵ\;f^{2}+ℵ{)}^{-1}}}+\;\sqrt{-2\;(\Omega \;ℵ\;f^{2}+ℵ{)}^{-1}}\;r\;\kappa \\ & \times (\frac{-\nu +\sqrt{4\kappa \;\zeta -\nu^{2}}\text{cot}\left(\frac{\sqrt{4\kappa \;\zeta -\nu^{2}}\;\eta }{2}\right)}{2\;\zeta }{)}^{-1})\times \;e^{\iota (\sigma \frac{x^{\beta }}{\beta }+\left(2\;\kappa \;\zeta \;r^{2}-\frac{1}{2}\;\nu^{2}r^{2}-\sigma^{2}\right)\frac{t^{\ell }}{\ell }+\Gamma )}. \end{array} \end{array}$$ (62)

$$\begin{array}{c} \begin{array}{cc} \psi_{14,2}\left(x,t\right)= & f(-\frac{\nu \;r}{ℵ\;(\Omega \;f^{2}+1)}\frac{1}{\sqrt{-2\;(\Omega \;ℵ\;f^{2}+ℵ{)}^{-1}}}+\;\sqrt{-2\;(\Omega \;ℵ\;f^{2}+ℵ{)}^{-1}}r\;\kappa \\ & \times (\frac{-\nu +\sqrt{4\kappa \;\zeta -\nu^{2}}\text{cot}\left(\frac{\sqrt{4\kappa \;\zeta -\nu^{2}}\;\eta }{2}\right)}{2\;\zeta }{)}^{-1})\times \;e^{\iota (\sigma \frac{x^{\beta }}{\beta }+\left(2\;\kappa \;\zeta \;r^{2}-\frac{1}{2}\;\nu^{2}r^{2}-\sigma^{2}\right)\frac{t^{\ell }}{\ell }+\Gamma )}. \end{array} \end{array}$$ (63)

(Set 2) if *ν*^2^ − 4*κζ* > 0 and *ζ* ≠ 0,

$$\begin{array}{c} \begin{array}{cc} \psi_{15,1}\left(x,t\right)= & (-\frac{\nu \;r}{ℵ\;(\Omega \;f^{2}+1)}\frac{1}{\sqrt{-2\;(\Omega \;ℵ\;f^{2}+ℵ{)}^{-1}}}+\;\sqrt{-2\;(\Omega \;ℵ\;f^{2}+ℵ{)}^{-1}}r\;\kappa \\ & \times (-\frac{\nu +\sqrt{\nu^{2}-4\kappa \;\zeta }\;\text{tan}\left(\frac{\sqrt{\nu^{2}-4\kappa \;\zeta }\;\eta }{2}\right)}{2\;\zeta }{)}^{-1})\times \;e^{\iota (\sigma \frac{x^{\beta }}{\beta }+\left(2\;\kappa \;\zeta \;r^{2}-\frac{1}{2}\;\nu^{2}r^{2}-\sigma^{2}\right)\frac{t^{\ell }}{\ell }+\Gamma )}. \end{array} \end{array}$$ (64)

$$\begin{array}{c} \begin{array}{cc} \psi_{15,2}\left(x,t\right)= & f(-\frac{\nu \;r}{ℵ\;(\Omega \;f^{2}+1)}\frac{1}{\sqrt{-2\;(\Omega \;ℵ\;f^{2}+ℵ{)}^{-1}}}+\;\sqrt{-2\;(\Omega \;ℵ\;f^{2}+ℵ{)}^{-1}}r\;\kappa \\ & \times (-\frac{\nu +\sqrt{\nu^{2}-4\kappa \;\zeta }\;\text{tan}\left(\frac{\sqrt{\nu^{2}-4\kappa \;\zeta }\;\eta }{2}\right)}{2\;\zeta }{)}^{-1})\times \;e^{\iota (\sigma \frac{x^{\beta }}{\beta }+\left(2\;\kappa \;\zeta \;r^{2}-\frac{1}{2}\;\nu^{2}r^{2}-\sigma^{2}\right)\frac{t^{\ell }}{\ell }+\Gamma )}. \end{array} \end{array}$$ (65)

**Or**

$$\begin{array}{c} \begin{array}{cc} \psi_{16,1}\left(x,t\right)= & (-\frac{\nu \;r}{ℵ\;(\Omega \;f^{2}+1)}\frac{1}{\sqrt{-2\;(\Omega \;ℵ\;f^{2}+ℵ{)}^{-1}}}+\;\sqrt{-2\;(\Omega \;ℵ\;f^{2}+ℵ{)}^{-1}}r\;\kappa \\ & \times (-\frac{\nu +\sqrt{\nu^{2}-4\kappa \;\zeta }\text{cot}\left(\frac{\sqrt{\nu^{2}-4\kappa \;\zeta }\;\eta }{2}\right)}{2\;\zeta }{)}^{-1})\times \;e^{\iota (\sigma \frac{x^{\beta }}{\beta }+\left(2\;\kappa \;\zeta \;r^{2}-\frac{1}{2}\;\nu^{2}r^{2}-\sigma^{2}\right)\frac{t^{\ell }}{\ell }+\Gamma )}. \end{array} \end{array}$$ (66)

$$\begin{array}{c} \begin{array}{cc} \psi_{16,2}\left(x,t\right)= & f(-\frac{\nu \;r}{ℵ\;(\Omega \;f^{2}+1)}\frac{1}{\sqrt{-2\;(\Omega \;ℵ\;f^{2}+ℵ{)}^{-1}}}+\;\sqrt{-2\;(\Omega \;ℵ\;f^{2}+ℵ{)}^{-1}}r\;\kappa \\ & \times (-\frac{\nu +\sqrt{\nu^{2}-4\kappa \;\zeta }\text{cot}\left(\frac{\sqrt{\nu^{2}-4\kappa \;\zeta }\;\eta }{2}\right)}{2\;\zeta }{)}^{-1})\times \;e^{\iota (\sigma \frac{x^{\beta }}{\beta }+\left(2\;\kappa \;\zeta \;r^{2}-\frac{1}{2}\;\nu^{2}r^{2}-\sigma^{2}\right)\frac{t^{\ell }}{\ell }+\Gamma )}. \end{array} \end{array}$$ (67)

(Set 3) if *ν*^2^ − 4*κζ* = 0 and *ζ* ≠ 0,

$$\begin{array}{c} \begin{array}{cc} \psi_{17,1}\left(x,t\right)= & (-\frac{\nu \;r}{ℵ\;(\Omega \;f^{2}+1)}\frac{1}{\sqrt{-2\;(\Omega \;ℵ\;f^{2}+ℵ{)}^{-1}}}+\;\sqrt{-2\;(\Omega \;ℵ\;f^{2}+ℵ{)}^{-1}}r\;\kappa \\ & \times (-\frac{2+\nu \;\eta }{2\;\zeta \;\eta }{)}^{-1})\times \;e^{\iota (\sigma \frac{x^{\beta }}{\beta }+\left(2\;\kappa \;\zeta \;r^{2}-\frac{1}{2}\;\nu^{2}r^{2}-\sigma^{2}\right)\frac{t^{\ell }}{\ell }+\Gamma )}. \end{array} \end{array}$$ (68)

$$\begin{array}{c} \begin{array}{cc} \psi_{17,2}\left(x,t\right)= & f(-\frac{\nu \;r}{ℵ\;(\Omega \;f^{2}+1)}\frac{1}{\sqrt{-2\;(\Omega \;ℵ\;f^{2}+ℵ{)}^{-1}}}+\;\sqrt{-2\;(\Omega \;ℵ\;f^{2}+ℵ{)}^{-1}}r\;\kappa \\ & \times (-\frac{2+\nu \;\eta }{2\;\zeta \;\eta }{)}^{-1})\times \;e^{\iota (\sigma \frac{x^{\beta }}{\beta }+\left(2\;\kappa \;\zeta \;r^{2}-\frac{1}{2}\;\nu^{2}r^{2}-\sigma^{2}\right)\frac{t^{\ell }}{\ell }+\Gamma )}. \end{array} \end{array}$$ (69)

## 4 Graphical explanation

In this section, we visually depict the physical characteristics of wave patterns in the studied dynamical systems through 3D, density, contour, and 2D graphical representations to display the exact optical solutions concerning wave velocity. We conduct physical simulations to obtain these solutions using appropriate values for the arbitrary parameters in the time-fractional coupled nonlinear Schrödinger equation. A modern software program, Maple ensures a clear and visually appealing presentation of the graphs.

**Fig 1:**
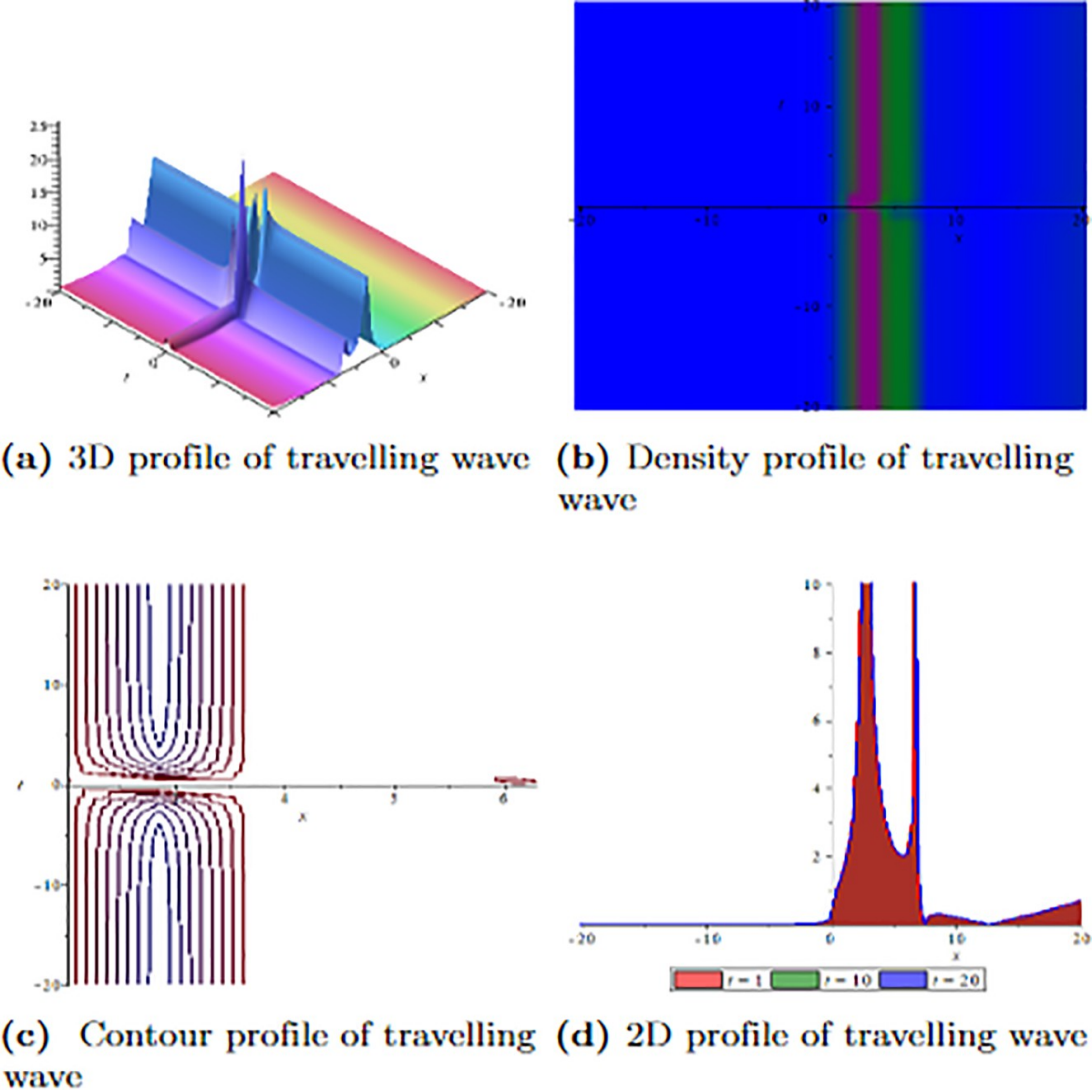
Graphical visualization of travelling wave for *ψ*_2,1_(*x*, *t*) with parameters, *δ* = 1.3, Γ = 0.25, ℵ = −1.25, *f* = 0.45, *β* = 0.5, *ϑ* = 0.75, *r* = 0.65, *v* = 0.9, *σ* = 1.36, *ℓ* = −0.63 and Ω = 1.25.

**Fig 2:**
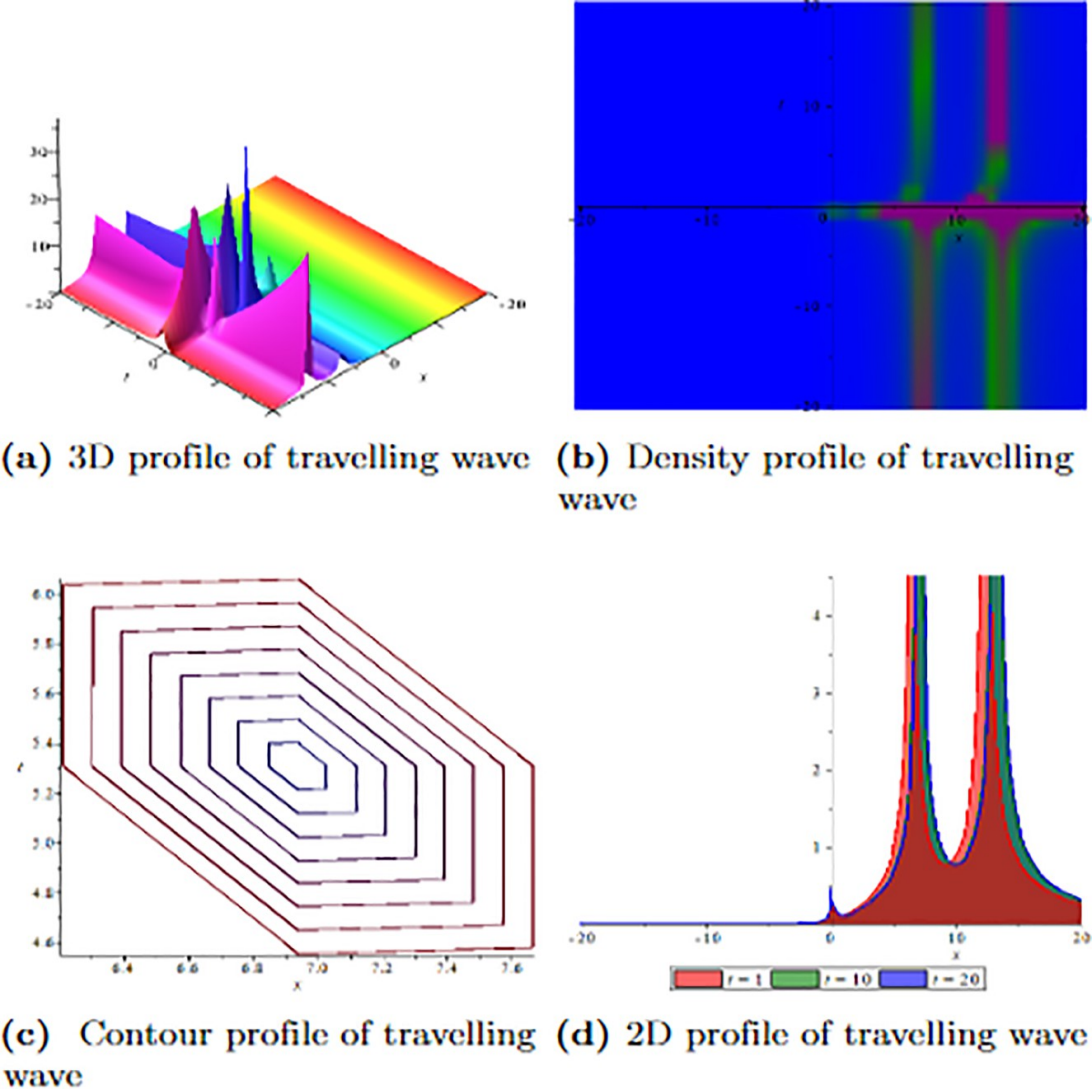
Graphical visualization of travelling wave for *ψ*_3,2_(*x*, *t*) with parameters, *δ* = 1.3, Γ = 0.25, ℵ = −1.25, *f* = 0.45, *β* = 0.5, *ϑ* = 0.75, *r* = 0.65, *v* = 0.2, *σ* = 1.36, *ℓ* = −0.62 and Ω = 1.25.

**Fig 3:**
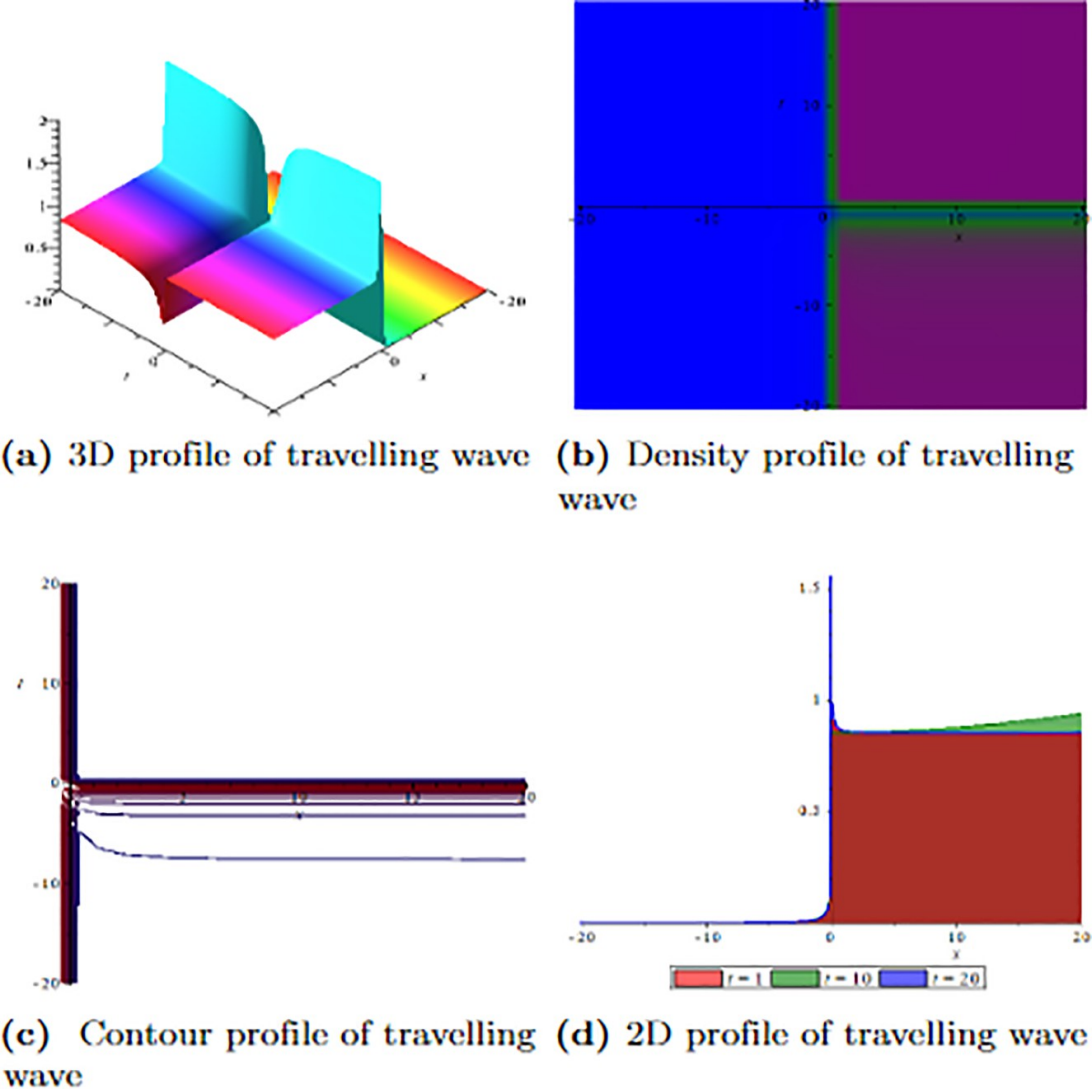
Graphical visualization of travelling wave for *ψ*_6,1_(*x*, *t*) with parameters, *δ* = 1.3, Γ = 0.25, ℵ = −1.25, *f* = 0.45, *β* = 0.3, *ϑ* = 0.75, *r* = 0.65, *v* = 0.9, *σ* = 1.36, *ℓ* = −1.25 and Ω = 1.25.

**Fig 4:**
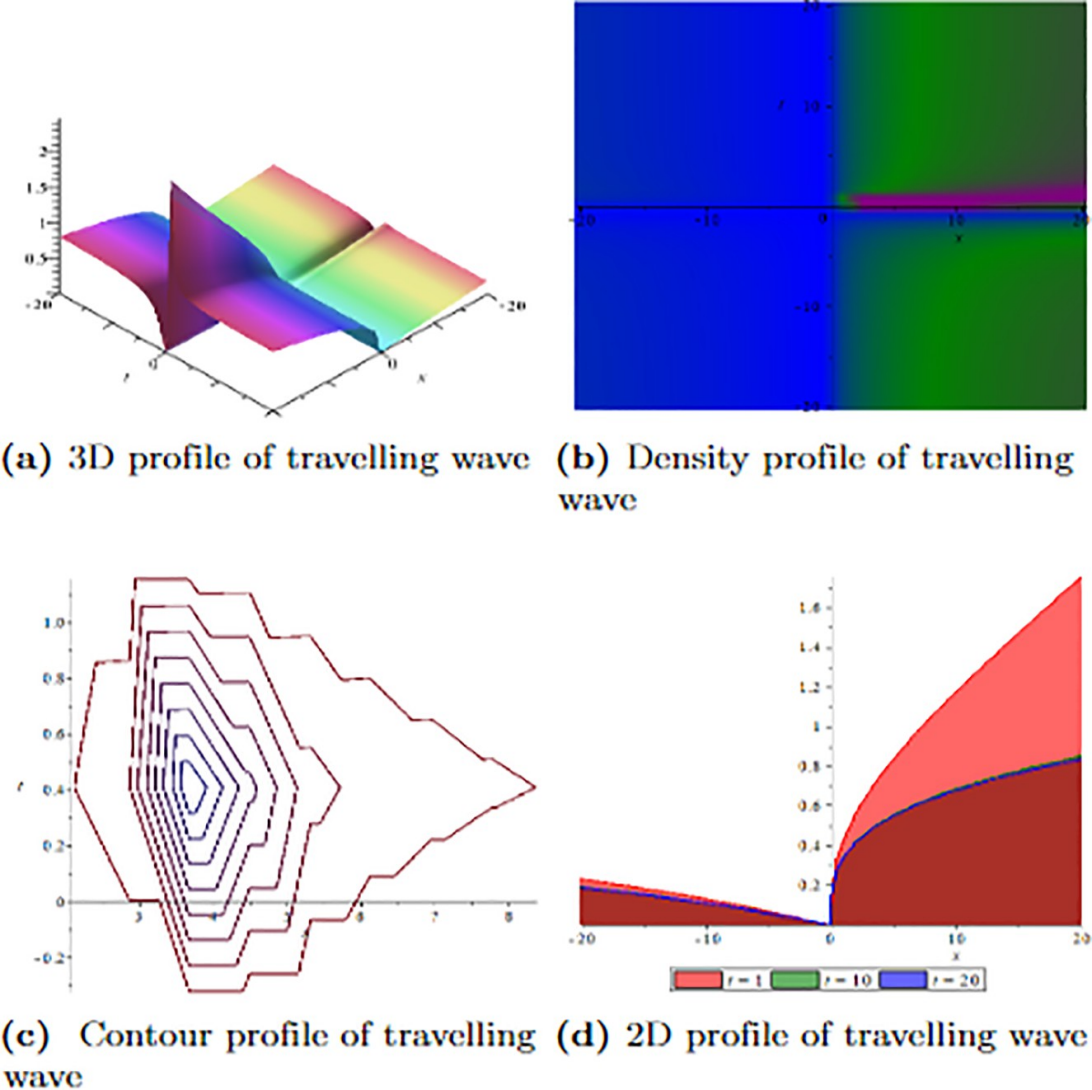
Graphical visualization of travelling wave for *ψ*_7,2_(*x*, *t*) with parameters, *δ* = 1.3, Γ = 1.25, ℵ = 1.5, *f* = 0.45, *c* = 2.5, *β* = −0.3, *ϑ* = 0.75, *r* = 0.65, *v* = 0.9, *σ* = 1.36, *ℓ* = −1.25 and Ω = 1.25.

Fig 1 depicts the wave velocity characteristics of the soliton solution, *ψ*_2,1_(*x*, *t*) with parametric values, *δ* = 1.3, Γ = 0.25, ℵ = −1.25, *f* = 0.45, *β* = 0.5, *ϑ* = 0.75, *r* = 0.65, *v* = 0.9, *σ* = 1.36, *ℓ* = −0.63 and Ω = 1.25, 3D profile exhibits combined dark-lump wave soliton within the intervals −20 ≤ *x* ≤ 20 and −20 ≤ *t* ≤ 20 and for more visualization contour and density graphs are plotted and dark periodic soliton observed while 2D shows a dark soliton. Fig 2 depicts the wave velocity characteristics of the soliton solution, *ψ*_3,2_(*x*, *t*) with parametric values, *δ* = 1.3, Γ = 0.25, ℵ = −1.25, *f* = 0.45, *β* = 0.5, *ϑ* = 0.75, *r* = 0.65, *v* = 0.2, *σ* = 1.36, *ℓ* = −0.62 and Ω = 1.25, 3D profile exhibits multiple dark-lump wave soliton within the intervals −20 ≤ *x* ≤ 20 and −20 ≤ *t* ≤ 20 and for more visualization contour and density graphs are plotted which shows a bright soliton while 2D shows a bright-periodic soliton. Fig 3 presents graphical representations of soliton solution *ψ*_6,1_(*x*, *t*) with parameters, *δ* = 1.3, Γ = 0.25, ℵ = −1.25, *f* = 0.45, *β* = 0.3, *ϑ* = 0.75, *r* = 0.65, *v* = 0.9, *σ* = 1.36, *ℓ* = −1.25 and Ω = 1.25, two dark-kink wave are evident in 3D within the intervals −20 ≤ *x* ≤ 20 and −20 ≤ *t* ≤ 20. The contour and density show dark wave behaviour, while the 2D profile represents the singular profile. In Fig 4, the 3D profile reveals a flat-kink with lump solitary wave for solution *ψ*_7,2_(*x*, *t*) and with the parametric values, *δ* = 1.3, Γ = 1.25, ℵ = 1.5, *f* = 0.45, *c* = 2.5, *β* = −0.3, *ϑ* = 0.75, *r* = 0.65, *v* = 0.9, *σ* = 1.36, *ℓ* = −1.25 and Ω = 1.25. Similarly, the contour plot exhibits bright solitons. At the same time, the 2D representation portrays a singular wave profile.

**Fig 5:**
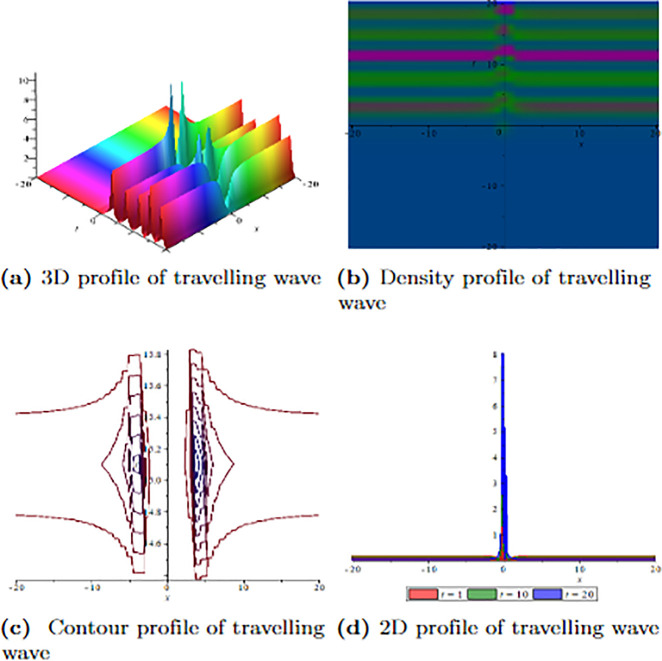
Graphical visualization of travelling wave for *ψ*_8,1_(*x*, *t*) with parameters, *ζ* = 1.3, Γ = 0.25, ℵ = 0.5, *f* = 0.45, *ν* = 0.5, *β* = −1.95, *κ* = 0.175, *r* = 0.65, *v* = −1.75, *σ* = 1.36, *ℓ* = 1.25 and Ω = 1.25.

**Fig 6:**
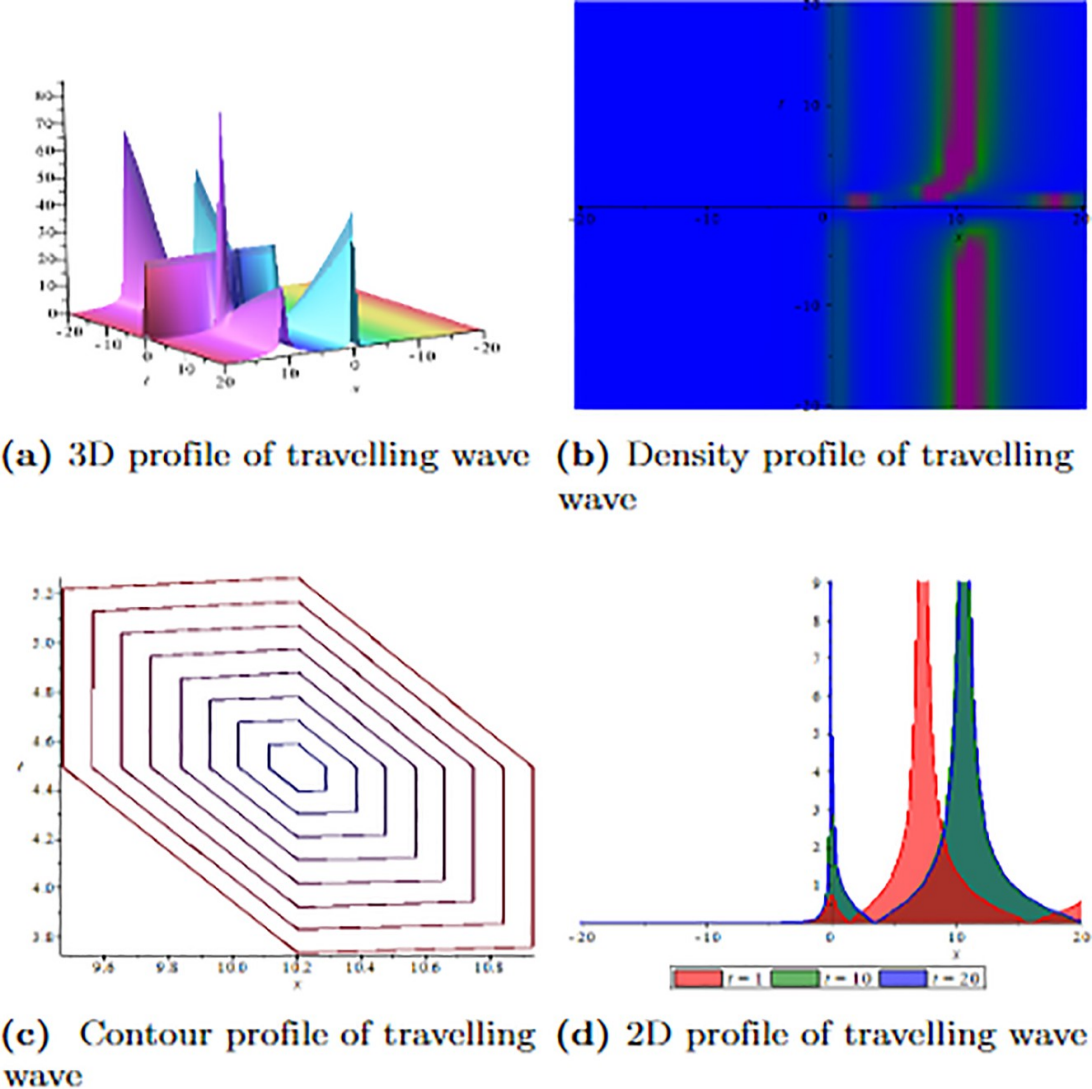
Graphical visualization of travelling wave for *ψ*_11,1_(*x*, *t*) with parameters, *ζ* = −2.3, Γ = 0.25, ℵ = 0.5, *f* = 0.45, *ν* = 0.5, *β* = 0.63, *κ* = 0.175, *r* = 0.65, *v* = 1.9, *σ* = 1.36, *ℓ* = −1.25 and Ω = 1.25.

**Fig 7:**
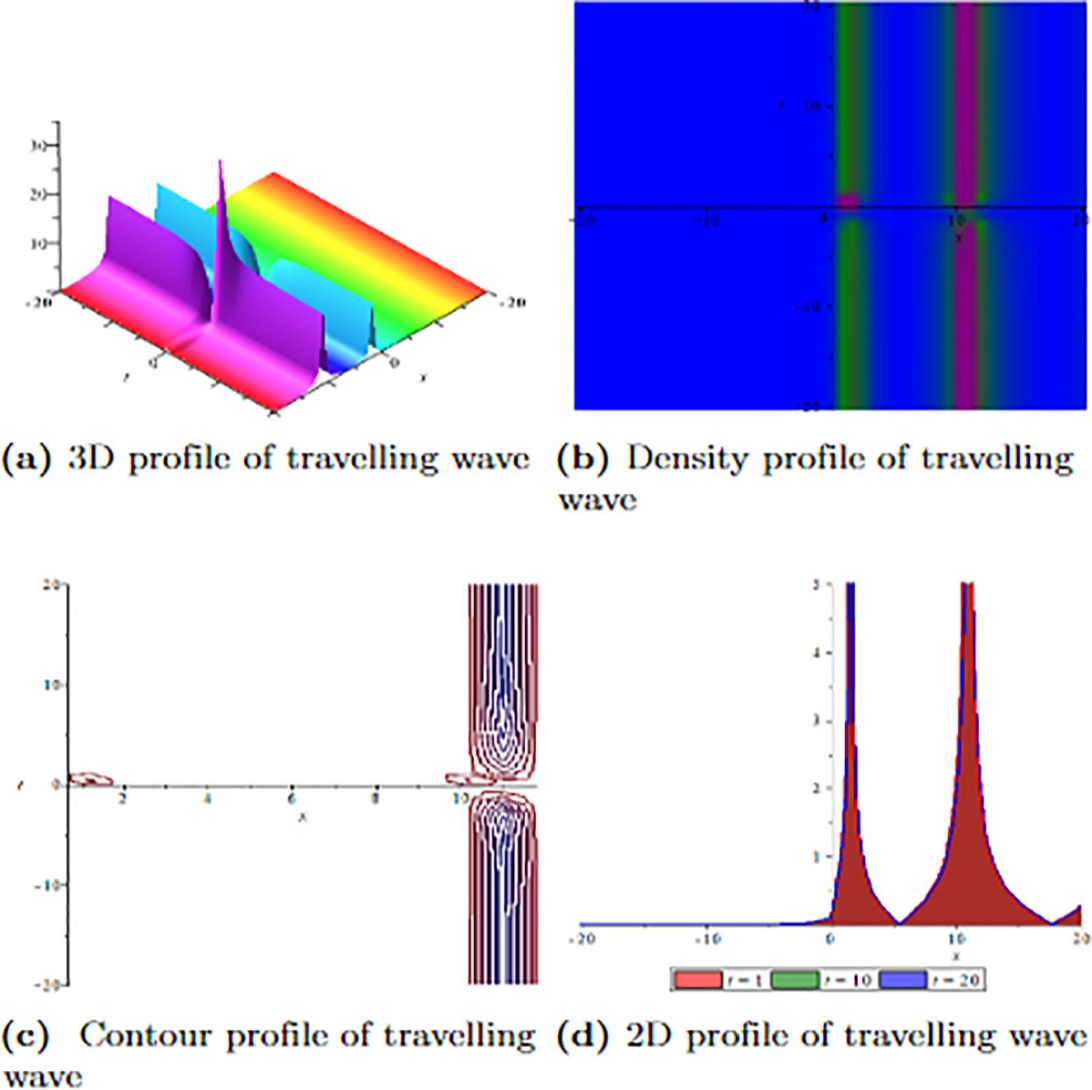
Graphical visualization of travelling wave for *ψ*_14,2_(*x*, *t*) with parameters, *ζ* = 1.3, Γ = 0.25, ℵ = 0.5, *f* = 0.45, *ν* = 0.5, *β* = 0.63, *κ* = 0.75, *r* = 0.65, *v* = 0.09, *σ* = 1.36, *ℓ* = −1.5 and Ω = 1.25.

**Fig 8:**
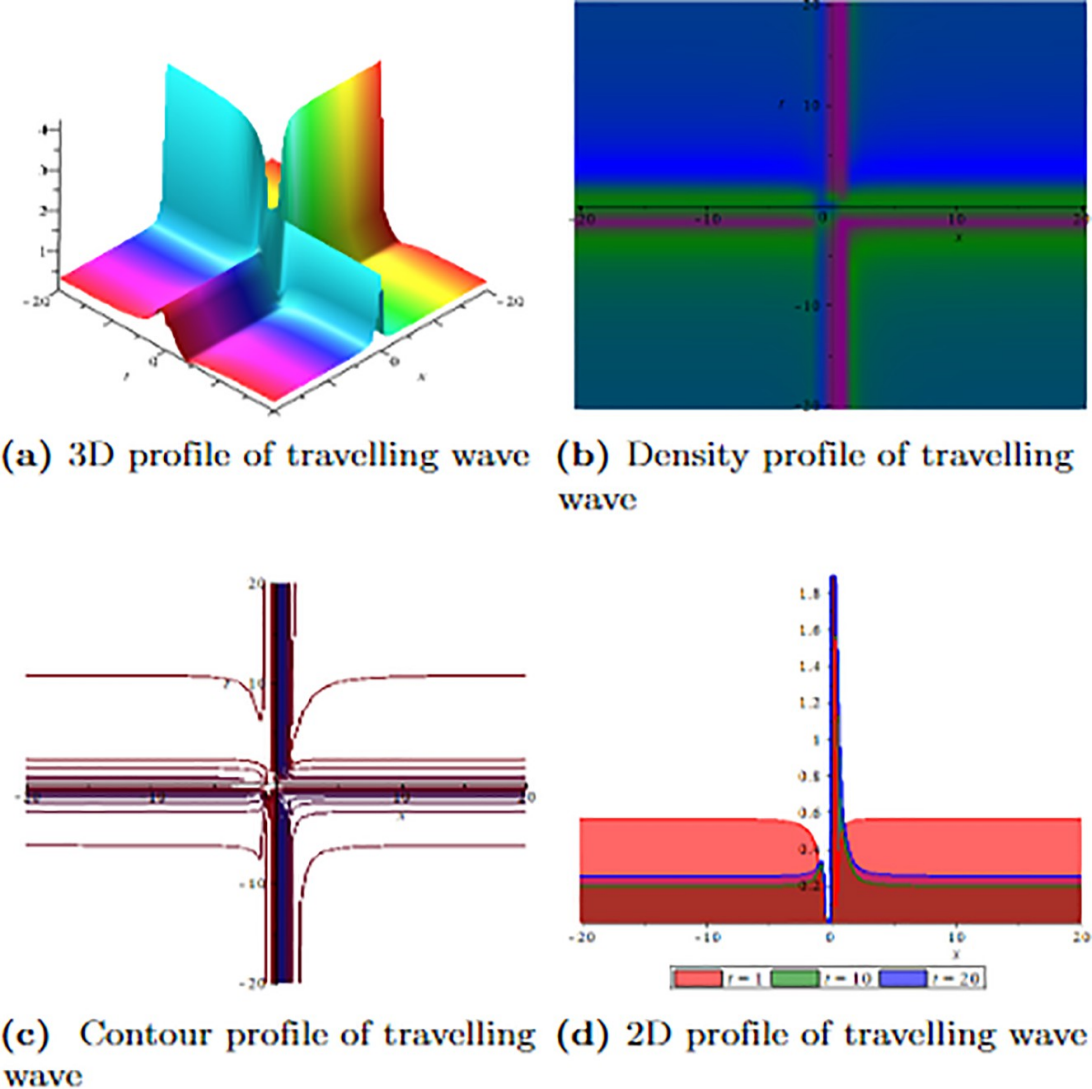
Graphical visualization of travelling wave for *ψ*_16,1_(*x*, *t*) with parameters, *ζ* = 1.3, Γ = 0.25, ℵ = 0.5, *f* = 0.45, *ν* = 0.5, *β* = −2.3, *κ* = 0.75, *r* = 0.65, *v* = 2.9, *σ* = 1.36, *ℓ* = −1.25 and Ω = 1.25.

In Fig 5, graphs are plotted for solution *ψ*_8,1_(*x*, *t*) with parameters, *ζ* = 1.3, Γ = 0.25, ℵ = 0.5, *f* = 0.45, *ν* = 0.5, *β* = −1.95, *κ* = 0.175, *r* = 0.65, *v* = −1.75, *σ* = 1.36, *ℓ* = 1.25 and Ω = 1.25 multiple U-shaped with lump-waves structure is observed in 3D while bright shows a bright-periodic and 2D shows a singular soliton. Fig 6 with parametric values, *ζ* = −2.3, Γ = 0.25, ℵ = 0.5, *f* = 0.45, *ν* = 0.5, *β* = 0.63, *κ* = 0.175, *r* = 0.65, *v* = 1.9, *σ* = 1.36, *ℓ* = −1.25 and Ω = 1.25, combined bright-dark with high amplitude soliton can be seen in 3D profile. In contrast, Fig 7 shows bright-dark with lump waves with parametric values, *ζ* = 1.3, Γ = 0.25, ℵ = 0.5, *f* = 0.45, *ν* = 0.5, *β* = 0.63, *κ* = 0.75, *r* = 0.65, *v* = 0.09, *σ* = 1.36, *ℓ* = −1.5 and Ω = 1.25, while bright and periodic-singular soliton in contour and 2D graphs, respectively. In Fig 8, graphs are plotted for soliton solution *ψ*_16,1_(*x*, *t*) with parameters, *ζ* = 1.3, Γ = 0.25, ℵ = 0.5, *f* = 0.45, *ν* = 0.5, *β* = −2.3, *κ* = 0.75, *r* = 0.65, *v* = 2.9, *σ* = 1.36, *ℓ* = −1.25 and Ω = 1.25. The graph transitions to a kink dark-periodic lump-wave solitary pattern, and the contour shows a dark-periodic soliton, while 2D shows a singular soliton.

The outcomes and justifications presented in this work are expected to significantly impact future research on nonlinear wave challenges in applied sciences. Our work has provided novel insights and approaches to addressing these challenges, and we believe that this will open up new avenues of research that can lead to further advancements in this field.

### 4.1 Comparative study and discussion

To show the uniqueness of our study and the efficiency of our methods, we compare our results with those found in most current literature in this section. Ahmed et al. [37] have used an extended tanh-expansion scheme to present the bright, dark, periodic, and singular solitons. In [38], Ali et al. used the F-modified expansion and unified techniques to obtain kink, periodic and singular soliton solutions. The q-homotopy analysis transform method is applied to obtain the analytical solutions [39]. The semi-inverse variational principle method and extended trial equation method are used in [40] to get analytical soliton solutions in the form of rational soliton, periodic soliton and hyperbolic soliton. This comparison highlights the unique contribution of our study in this regard and shows the variety of soliton solutions in various investigations.

While some of our findings, like kink, dark, and periodic solitons, are consistent with prior research, the rest of our results unveil entirely novel soliton solutions. These consist of dark-lump wave soliton, Multiple dark-lump wave soliton, two dark-kink solitons, flat kink-lump wave, multiple U-shaped with lump wave, combined bright-dark with high amplitude lump wave, bright-dark with lump wave. This evident difference between our solutions and those already published draws attention to the uniqueness and novelty of our study with efficient methods.

Furthermore, using our suggested techniques has made finding many solutions more straightforward. The wide variety and breadth of soliton solutions examined in this study will pique readers’ interest. Moreover, our study provides a dynamic evaluation of the suggested model through chaotic analysis. This added layer of this investigation improves the research’s attractiveness by offering more profound insights into the behaviour of the researched model under varied settings. It advances our comprehension of the phenomena under study.

While the GPRE and MAE methods have successfully solved various types of differential equations, their effectiveness may vary depending on the complexity of the equation. In cases where numerical or qualitative analyses are needed, supplementary methods might be required. Additionally, equations with highly nonlinear or intricate structures may present challenges for these methods.

## 5 Dynamical study of TFCNLSE

The Galilean transformation process can introduce chaotic analysis to the time-fractional coupled nonlinear Schrödinger equation within the dynamical system. Therefore, the resulting system that is in motion is as follows:

$$\begin{array}{c} \left\{ \begin{array}{c} \frac{d\;\psi }{d\zeta }=W,\\ \frac{dW}{d\eta }=-\frac{\left(ℵ+ℵ\;\Omega \;f^{2}\right)\psi_{1}^{3}}{r^{2}}+\frac{\left(\sigma +\omega \right)\psi_{1}}{r^{2}}, \end{array}\right. \end{array}$$ (70)

### 5.1 Chaos analysis

In nonlinear science, chaos theory holds significant importance and finds widespread applications across natural sciences, encompassing medical science, fluid dynamics, optics, plasma physics, and material physics. This section explores quasi-periodic chaotic, quasi-periodic, and periodic resonant oscillations concerning the soliton waves within a perturbed dynamical system Eq (25). Introducing a perturbation factor ϒ_0_, cos(*ϰ*, *t*) via Eq (70) significantly enhances the precision of our simulation tools. These tools incorporate phase portrait analysis in 3D, time series analysis, and Poincaré section, collectively enhancing our comprehension of the nonlinear periodic solitary wave dynamics.

$$\begin{array}{c} \left\{ \begin{array}{c} \frac{d\;\psi }{d\zeta }=W,\\ \frac{dW}{d\eta }=-\frac{\left(ℵ+ℵ\;\Omega \;f^{2}\right)\psi_{1}^{3}}{r^{2}}+\frac{\left(\sigma +\omega \right)\psi_{1}}{r^{2}}+{ϒ}_{0}\;\text{cos}\left(\varkappa ,t\right). \end{array}\right. \end{array}$$ (71)

The nonlinear behaviour of the dynamical system is significantly affected by the perturbation factor [45] and the selection of initial conditions. Consequently, we investigate different parameter ranges and systematically adjust the amplitudes *Υ*_0_ and angular *ϰ* to analyze the system’s dynamics.

**Fig 9:**
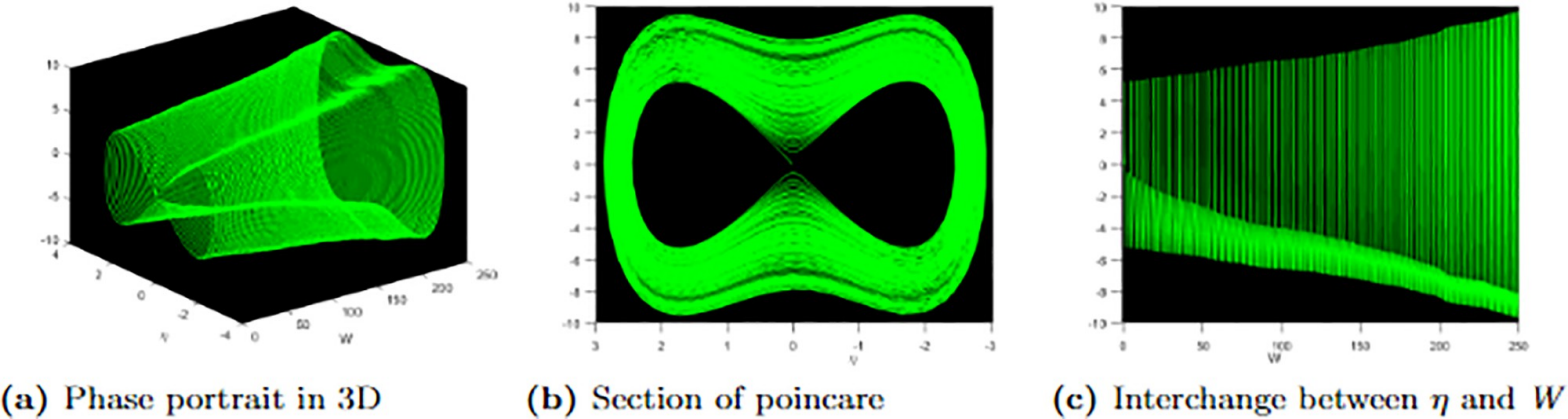
Profile of periodic with parametric values, ℵ = 0.03, Ω = 0.05, *f* = 0.06, *σ* = 0..08, *ω* = 0.08, *r* = 0.07 and the perturbation term.

Fig 9 exhibits 3D, Poincare, and time series graphs corresponding to the parameters *ϰ* = 0.09, Υ_0_ = 0.002, and an initial condition of (0, 0.25). These graphical representations visually depict the periodic behaviour observed in the perturbed system within the dynamical plane, as described by Eq (71).

**Fig 10:**
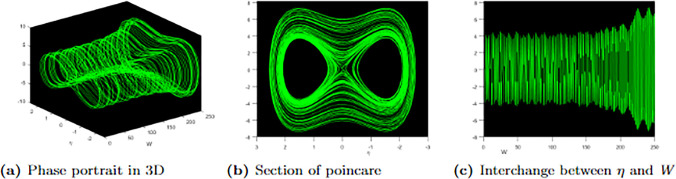
Profile of quasi-periodic with parametric values, ℵ = 0.03, Ω = 0.05, *f* = 0.06, *σ* = 0.01, *ω* = 0.06, *r* = 0.07 and the perturbation term.

On the other hand, Fig 10 displays 3D, Poincare, and time series graphs for the same initial condition (0, 0.25) but with different parameters *ϰ* = 2.9 and Υ_0_ = 0.5. In this case, the perturbed system exhibits quasi-periodic behaviour, following the dynamics described by Eq (71).

**Fig 11:**
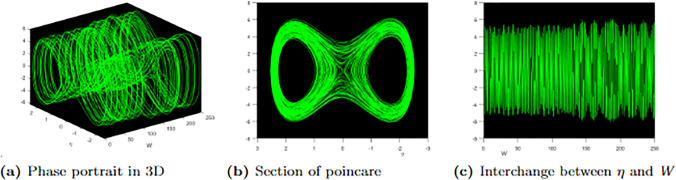
Profile of quasi-periodic chaotic with parametric values, ℵ = 0.03, Ω = 0.05, *f* = 0.06, *σ* = 0.01, *ω* = 0.08, *r* = 0.07 and the perturbation term.

The graphical representations in Fig 11 illustrate the presence of patterns that display some periodicity characteristics but with variations in the time or amplitude of each repetition, highlighting the quasi-periodic nature of the system. Moving on to Fig 11, we observe 3D, Poincare, and time series graphs at initial conditions (0, 0.25). The perturbed system, governed by Eq (71), exhibits quasi-periodic chaotic behaviour when *ϰ* = 4.1 and Υ_0_ = 1.2.

## 6 Conclusion

This study explores a wide range of travelling wave soliton solutions to the nonlinear time-fractional coupled nonlinear Schrödinger equation using the generalized projective Riccati equation and modified auxiliary equation methods, revealing explicit solitonic structures. Novel soliton solutions, including combined dark-lump wave solitons, multiple dark-lump wave solitons, two dark-kink solitons, flat kink-lump waves, multiple U-shaped with lump waves, combined bright-dark with high amplitude lump waves, and kink dark-periodic solitons, are derived. Graphical illustrations in three dimensions, two dimensions, density, and contour graphs enhance understanding of these waves. Additionally, chaotic behaviour analysis of the dynamical system under various initial conditions and parameters is conducted. These obtained solutions offer valuable insights into the behaviour of waves in nonlinear media and hold significant implications for various fields. The presented methodologies yield comprehensive results in four formats, showcasing higher efficiency. Such findings can empower researchers and professionals to apply complex nonlinear systems to linear equations and various scientific fields more effectively. This examination sheds light on the equation’s configuration and provides a valuable tool for validating the model’s findings under consideration.

## Supporting information

S1 Dataset (PDF)
