# Correction: Analyzing chaos and superposition of lump waves with other waves in the time-fractional coupled nonlinear Schördinger equation

**DOI:** 10.1371/journal.pone.0313333

**Published:** 2024-10-31

**Authors:** Sheikh Zain Majid, Muhammad Imran Asjad, Muhammad Bilal Riaz, Taseer Muhammad

The word "Schrödinger" is misspelled in the article title. The correct title is: Analyzing chaos and superposition of lump waves with other waves in the time-fractional coupled nonlinear Schrödinger equation. The correct citation is: Majid SZ, Asjad MI, Riaz MB, Muhammad T (2024) Analyzing chaos and superposition of lump waves with other waves in the time-fractional coupled nonlinear Schrödinger equation. PLoS ONE 19(8): e0304334. https://doi.org/10.1371/journal.pone.0304334.

There is an error in affiliation 3 for author Muhammad Bilal Riaz. The correct affiliation 3 is: It4Innovations, VSB-Technical University of Ostrava, Ostrava, Czech Republic.

The funding statement is incorrect. The correct funding statement is as follows: This research received financial support from the European Union under the REFRESH–Research Excellence for Regional Sustainability and High-Tech Industries project, number CZ.10.03.01/00/22_003/0000048, through the Operational Programme Just Transition. The authors also extend their appreciation to the Deanship of Research and Graduate Studies at King Khalid University, Abha, Saudi Arabia, for funding this work through a Small Research Project under grant number RGP.1/141/45.
