# Supplementary material for: Analyzing chaos and superposition of lump waves with other waves in the time-fractional coupled nonlinear schördinger equation
Source: PLoS One. 2024 Aug 28;19(8):e0304334. doi: 10.1371/journal.pone.0304334 (PMC11356411; doi:10.1371/journal.pone.0304334)
Supplement: S1 Dataset — (PDF) [file pone.0304334.s001.pdf]

Manuscript ID: PONE-D-24-08941R1
Title: Analyzing chaos and superposition of lump waves with other waves in the time-fractional coupled nonlinear schördinger equation

---

1) The values behind the means, standard deviations, and other measures reported

Model: The time fractional coupled nonlinear Schr¨odinger equation, which explains the interaction between modes in nonlinear optics and Bose-Einstein condensation.

Methods: we are using two advanced and powerful techniques to solve the non-linear model and obtain the new solitary wave solutions.

- Generalized projective Riccati equation method.
- Modified auxiliary equation method.

To make it more flavorful we add a Dynamical investigation.

- Chaos analysis.

Software: we use three different software.

- Overleaf: for writing (https://www.overleaf.com/project)
- Mathematica: for plotting graphs (https://www.wolfram.com/mathematica/online/)
- Maple: for calculations (https://www.maplesoft.com/products/Maple/)

---

2) The values used to build graphs for soliton solutions using generalized projective Riccati equation method.

Graph values:

In "Figure 1", we express the solutions of $\psi 2,1(x, t)$ with parameters, $\delta = 1.3$, $\Gamma = 0.25$, $\aleph = -1.25$, $f = 0.45$, $\beta = 0.5$, $\theta = 0.75$, $r = 0.65$, $v = 0.9$, $\sigma = 1.36$, $\ell = -0.63$ and $\Omega = 1.25$.

In "Figure 2", we express the travelling wave for $\psi 3,2(x, t)$ with parameters, $\delta = 1.3$, $\Gamma = 0.25$, $\aleph = -1.25$, $f = 0.45$, $\beta = 0.5$, $\theta = 0.75$, $r = 0.65$, $v = 0.2$, $\sigma = 1.36$, $\ell = -0.62$ and $\Omega = 1.25$.

In "Figure 3", we express the solutions of $\psi 6,1(x, t)$ with parameters, $\delta = 1.3$, $\Gamma = 0.25$, $\aleph = -1.25$, $f = 0.45$, $\beta = 0.3$, $\theta = 0.75$, $r = 0.65$, $v = 0.9$, $\sigma = 1.36$, $\ell = -1.25$ and $\Omega = 1.25$.

In "Figure 4", we express the travelling wave $f\psi 7,2(x, t)$ with parameters, $\delta = 1.3$, $\Gamma = 1.25$, $\aleph = 1.5$, $f = 0.45$, $c = 2.5$, $\beta = -0.3$, $\theta = 0.75$, $r = 0.65$, $v = 0.9$, $\sigma = 1.36$, $\ell = -1.25$ and $\Omega = 1.25$.

3) The values used to build graphs for soliton solutions using modified auxiliary equation method.

Graph values:

In "Figure 5", we express the solutions of travelling wave for $\psi 8,1(x, t)$ with parameters, $\zeta = 1.3$, $\Gamma = 0.25$, $\aleph = 0.5$, $f = 0.45$, $\nu = 0.5$, $\beta = -1.95$, $\kappa = 0.175$, $r = 0.65$, $v = -1.75$, $\sigma = 1.36$, $\ell = 1.25$ and $\Omega = 1.25$.

In “Figure 6”, we express the travelling wave for ψ11,1(x, t) with parameters, $\zeta = -2.3$, $\Gamma = 0.25$, $\aleph = 0.5$, $f = 0.45$, $\nu = 0.5$, $\beta = 0.63$, $\kappa = 0.175$, $r = 0.65$, $v = 1.9$, $\sigma = 1.36$, $\ell = -1.25$ and $\Omega = 1.25$

In “Figure 7”, we express the solutions of ψ14,2(x, t) with parameters, $\zeta = 1.3$, $\Gamma = 0.25$, $\aleph = 0.5$, $f = 0.45$, $\nu = 0.5$, $\beta = 0.63$, $\kappa = 0.75$, $r = 0.65$, $v = 0.09$, $\sigma = 1.36$, $\ell = -1.5$ and $\Omega = 1.25$

In “Figure 8”, we express the travelling wave ψ16,1(x, t) with parameters, $\zeta = 1.3$, $\Gamma = 0.25$, $\aleph = 0.5$, $f = 0.45$, $\nu = 0.5$, $\beta = -2.3$, $\kappa = 0.75$, $r = 0.65$, $v = 2.9$, $\sigma = 1.36$, $\ell = -1.25$ and $\Omega = 1.25$

4) Chaotic analysis

In chaotic analysis “Figs 9-11” describes the Galilean transformation process can introduce chaotic analysis to the time-fractional coupled nonlinear Schrodinger equation within the dynamical system.

In “Figure 9”, Profile of periodic with parametric values, $\aleph = 0.03$, $\Omega = 0.05$, $f = 0.06$, σ = 0..08, $\omega = 0.08$, $r = 0.07$ and the perturbation term, $x = 0.09$, $Y0 = 0.002$.

In “Figure 10”, Profile of Quasi-periodic with parametric values, $\aleph = 0.03$, $\Omega = 0.05$, $f = 0.06$, $\sigma = 0.01$, $\omega = 0.06$, $r = 0.07$ and the perturbation term, $x = 2.9$ and $Y0 = 0.5$.

In “Figure 11”, Profile of Quasi-periodic chaotic with parametric values, $\aleph = 0.03$, $\Omega = 0.05$, $f = 0.06$, $\sigma = 0.01$, $\omega = 0.08$, $r = 0.07$ and the perturbation term, $x = 4.1$ and $Y0 = 1.2$.
